# Hybridization With an Invasive Plant of *Xanthium strumarium* Improves the Tolerance of Its Native Congener *X. sibiricum* to Cadmium

**DOI:** 10.3389/fpls.2021.696687

**Published:** 2021-07-29

**Authors:** Chenyang Xue, Yingmei Gao, Bo Qu, Peidong Tai, Cheng Guo, Wenyue Chang, Guanghui Zhao

**Affiliations:** ^1^College of Biological Technology, Shenyang Agricultural University, Shenyang, China; ^2^Liaoning Key Laboratory of Biological Invasions and Global Changes, Shenyang Agricultural University, Shenyang, China; ^3^Key Laboratory of Pollution Ecology and Environmental Engineering, Institute of Applied Ecology, Chinese Academy of Sciences, Shenyang, China; ^4^Liaoning Shihua University, Fushun, China; ^5^Shenyang Academy of Environmental Sciences, Shenyang, China

**Keywords:** *Xanthium strumarium*, *Xanthium sibiricum*, invasive plants, hybridization, Cd pollution, transcriptome

## Abstract

Hybridization is one of the important factors influencing the adaptive evolution of invasive plants. According to previous studies, hybridization with an invasive plant reduces the adaptability of its native congener to environment. However, in this study, the hybridization with an invasive plant of *Xanthium strumarium* (LT) improves the tolerance and accumulation of its native congener *Xanthium sibiricum* (CR) to cadmium (Cd). Under Cd stress, *X. sibiricum*♀ × *X. strumarium*♂ (ZCR) showed higher biomass and Cd accumulation. Compared with CR, ZCR has longer vegetative and reproductive growth time. Moreover, ZCR adopted more reasonable biomass allocation strategy. ZCR increased the proportion of reproductive allocation and ensured its own survival with the increase of Cd stress. Furthermore, ZCR increased the translocation of Cd to aboveground parts and changed the distribution of Cd. A large amount of Cd is stored in senescent leaves and eliminated from the plant when the leaves fall off, which not only reduces the Cd content in the plant, but also reduces the toxicity of Cd in the normal leaves. Transcriptome analysis shows a total of 2055 (1060 up and 995 down) differentially expressed genes (DEGs) were detected in the leaves of Cd-stressed ZCR compared with CR, while only 792 (521 up and 271 down) were detected in *X. strumarium*♀ × *X. sibiricum*♂ (ZLT) compared with LT. A large number of DGEs in ZCR and ZLT are involved in abscisic acid (ABA) synthesis and signal transduction. The genes induced by ABA in ZCR, including CNGC5/20, CPK1/28, CML, PTI1-like tyrosine-protein kinase 3, respiratory burst oxidase homolog protein C, and WRKY transcription factor 33 were found differentially expressed compared CR. carotenoid cleavage dioxygenase 4, NCED1/2, phytoene synthase 2, and CYP707A involved in ABA synthesis and decomposition in ZLT were found differentially expressed compared LT. We speculated that ABA played an important role in Cd transportation of hybrids and Cd distribution in senescent and normal leaves. The results demonstrate that hybridization with an invasive plant improves the adaptability of the hybrid to Cd stress and may enhance the extinction risk of native congener in pollution environment.

## Introduction

Large quantities of heavy metals have been discharged into the environment over the last three decades, coinciding with rapid industrialization and urbanization in China (Zhao et al., 2014; Jiang et al., 2020). Increasing soil heavy metal pollution has seriously affected the yield and quality of crops, and these pollutants are being transmitted upwards through food chains and food webs, affecting the health of animals and humans (Wachirawongsakorn, 2016; Liu et al., 2017).

As the world economy continues to change and human activities increased, biological invasion has become a global issue (Xue et al., 2018; Frost et al., 2019). Invasive plants tend to extract pollutants beyond their metabolic needs and exhibit desirable characteristics of hyperaccumulators, such as high growth rates and biomass (Pyšek et al., 2009; Parepa et al., 2013), high heavy metal extraction ability (Newete et al., 2016; Michalet et al., 2017), high tolerance to heavy metals (Wei et al., 2018; Liu et al., 2019). Current research on invasive plants as remediation materials mainly focuses on their tolerance and enrichment to a single pollutant in the environment (Sricoth et al., 2018; Wei et al., 2018; Huang et al., 2019; Liu et al., 2019; Kumwimba et al., 2020), ignoring the potential impact of the interaction between invasive plants and environmental pollution on invasive plants and its native congener plants.

Hybridization is one of the important means for adaptive evolution of invasive plants, and plays an important role in the introduction, establishment, and spread of invasive species (Facon et al., 2006; Lavergne and Molofsky, 2007), and can produce new genotypes with the combination of various characteristics (Lexer et al., 2003; Prentis et al., 2008). Hybridization can eliminate the detrimental mutations in the population, overcome self-incompatibility, reduce genetic bottlenecks and genetic load, and enhance species invasiveness (Rieseberg et al., 2003). As a result, hybrids usually demonstrate greater fitness and wider ecological tolerances than their parents. Such hybrids can harm native species through the loss of both genetic diversity and of locally adapted populations (Ellstrand and Schierenbeck, 2000). Aggressive hybrid can also result in reduced growth and even the extirpation of native species populations (Sun et al., 2015). As Chinese industrialization and urbanization develop and economic globalization further strengthens, the link between environmental pollution and biological invasion become increasingly close (Shackleton et al., 2019; Wu and Ding, 2019). Therefore, a better understanding of the combined impacts of hybridization and heavy metals on invasive and its native congener plants is crucial for the rational use and control of invasive plants (Shackleton et al., 2019; Rai and Singh, 2020).

*Xanthium strumarium* (LT), an annual herb belonging to the family Compositae and native to North America, has high stress resistance, fast growth, and large biomass, and it has become one of the most invasive plants in Northeast China and Xinjiang (Xun et al., 2017; Lin et al., 2018). *Xanthium sibiricum* (CR) is a common Compositae weed species, 20–90 cm high, flowering July–August, fruit September–October, the bracts of CR with hook-like hard spines, often attached to livestock and human body. It is fruit can be used as ink, soap, felt raw materials, but also can be used to make hard oil and lubricants. In addition, the fruit can be used as medicine. Hybridization has been demonstrated between LT and CR in nature through morphological structure analysis (Xue et al., 2018) and molecular experiments (Xue et al., 2020). High-throughput transcriptomic analysis, for example RNA-seq analysis based on Illumina sequencing technology, can help to identify potential biomarker genes and unravel underlying molecular mechanisms, and RNA-seq analysis needs no reference genome (Shi Y. L. et al., 2019; Yi et al., 2020). Therefore, this study was designed to: (1) assess the effect of hybridization on the growth and tolerance of LT and CR under Cd stress; (2) explore the possible mechanisms by transcriptomics analysis of the leaves of LT, CR, *X. sibiricum*♀ × *X. strumarium*♂ (ZCR), and *X. strumarium*♀ × *X. sibiricum*♂ (ZLT). These data can be provided reference in-depth study of the invasion mechanism and prevention of invasive plants.

## Materials and Methods

### Plant Materials Growth Conditions and Treatments

*Xanthium strumarium* and CR were collected from two populations (Supplementary Figure 1) along Hunhe River (123°7′E, 41°37′ N, HR) and Dalinghe River (121°34′ E, 41°04′ N, DR) in Shenyang City, Liaoning Province. The scientific research base of Shenyang Agricultural University in Liaoning Province was selected for the artificial self-pollination and hybridization tests of LT and CR. The healthy inflorescences of LT and CR were selected for bagging treatment at flowering stage, respectively (Supplementary Figure 2). The stamens of LT and CR used for hybridization were removed at the flowering stage, pollinated when the stigma was feathery and shiny, and the inflorescences after pollination were isolated by bagging (Supplementary Figure 3). After flowering stage, all the bags were removed. After the fruits were naturally mature, they were collected separately, air-dried in a ventilated place, and stored at room temperature for further use. In order to ensure that the test results are not disturbed by human selection, LT, CR, ZLT, and ZCR obtained from the two populations are mixed, respectively (Ni et al., 2014; Sun et al., 2015; Li et al., 2016; Xie et al., 2020; Zhang Q. L. et al., 2020).

*Xanthium strumarium* and CR have dimorphic seeds (Supplementary Figure 4). The lower seeds were selected as materials in this experiment in order to avoid unreliability and inconsistency of the results under the same conditions (Wu et al., 2009). After the seedlings were grown in trays in the glasshouse for 2 weeks, plants of the same size were selected and moved into the pots (27 cm, inner diameter 25 cm, 5 kg of soil/pot, one seedling per pot) on the 1st June. The four treatments with analytical grade CdCl_2_⋅2.5H_2_O were applied at concentrations of 0, 2.5, 5, 10 mg kg^–1^ (Zhang et al., 2014, 2018; Liu et al., 2016, 2019), corresponding to CK, C1, C2, and C3. The CdCl_2_⋅2.5H_2_O solution was added to the pots in a liquid state, fully mixed with the soil, and equilibrated for 2 weeks. The final cadmium concentrations in the soils were CK: 0.14 ± 0.01 mg kg^–1^, C1: 2.56 ± 0.14 mg kg^–1^, C2: 5.14 ± 0.01 mg kg^–1^, C3: 10.18 ± 0.18 mg kg^–1^, A total of 96 pots (4 species × 4 Cd treatments × 6 replicates) were used in greenhouses. The physical properties of potting soil were as follows: pH = 7.02, organic matter content = 12.84 g kg^–1^, total nitrogen content = 0.14 g kg^–1^, total phosphorus content = 0.79 g kg^–1^, the available soil nitrogen content = 59.33 mg kg^–1^, and the available soil phosphorus content = 42.85 mg kg^–1^. The vegetative reproductive stage of plants is from the abscission of two cotyledons to the initial flowering stage and the reproductive stage is from the initial flowering stage to the last flowering stage (Sherry et al., 2007; Shen et al., 2016). During the last flowering stage, each plant was collected at each treatment concentration. During the experiment, plants were irrigated daily with tap water (without the Cd^2+^/fertilizer) to compensate 70% water holding capacity of the soil.

Seed disinfection and germination of CR, LT, ZCR, and ZLT see our previous research (Xue et al., 2020). Seeds germinated were moved into quartz sand containing 1/2 Murashige and Skoog (MS) nutrient solution for 1 week. Then the same growing seedlings were moved into brown flasks (Figure 3) containing 100 μmol/L CdCl_2_ for 5 days in constant temperature incubator (25°C, 16 h light; 20°C, 8 h dark) (Zhang Q. L. et al., 2020). Fresh leaves were treated with liquid nitrogen and stored at −80°C.

### Determination of Plant Biomass and Cd Concentration in Pot Experiment

After the harvesting, the plants were washed with an aqueous solution of EDTA (5 mM) and Tris–HCl (pH = 6), followed by rinsing with distilled water three times, after which plants were divided into roots, stems, leaves, and inflorescence. Each part was transferred to a separate paper envelope, treated with 120°C for 15 min, dried in an oven at 80°C for 48 h, and weighed. Leaf litter (including withered and fallen leaves) was collected in order to avoid the loss of biomass and metal ions. A total of 0.2 g samples (root, steam, and leave) were weighed and digested by nitric acid-perchloric acid-hydrogen peroxide mixture (Volume ratio = 6:2:1) in a microwave digestion apparatus (MARS6 microwave digestion apparatus, CEM, United States) to determine Cd concentration using the Hitachi Z-2000 atomic absorption spectrophotometer (Hitachi High-Technologies Corporation, Tokyo, Japan).

The bioaccumulation factor (BCF) and translocation factor (TF) of CR, ZCR, ZLT, and LT were calculated in order to analyze the extraction and transport capacity of the plants for Cd (Tanhan et al., 2007). These two factors were calculated as follows:


$$\mathrm{BCF}=\frac{\mathrm{Concentration}\,\mathrm{of}\,\mathrm{metal}\,\mathrm{in}\,\mathrm{shoots}\,\left(\mathrm{mg}\cdot {\text{kg}}^{-1}\right)}{\mathrm{Concentration}\,\mathrm{of}\,\mathrm{metal}\,\mathrm{in}\,\mathrm{soil}\,\left(\mathrm{mg}\cdot {\text{kg}}^{-1}\right)}\,\mathrm{and}$$

$$\mathrm{TF}=\frac{\mathrm{Concentration}\,\mathrm{of}\,\mathrm{metal}\,\mathrm{in}\,\mathrm{shoots}\,\left(\mathrm{mg}\cdot {\text{kg}}^{-1}\right)}{\mathrm{Concentration}\,\mathrm{of}\,\mathrm{metal}\,\mathrm{in}\,\mathrm{roots}\,\left(\mathrm{mg}\cdot {\text{kg}}^{-1}\right)}$$

### RNA Isolation, Library Construction, *de novo* Assembly and Functional Annotation for Transcriptome Sequencing

Total RNA was isolated using the Trizol Reagent (Invitrogen Life Technologies), after which the concentration, quality, and integrity were determined using a NanoDrop Spectrophotometer (Thermo Fisher Scientific, Waltham, MA, United States). Oligo (dT) magnetic beads were used to enrich mRNA with a polyA structure in total RNA and disrupt RNA to 300–400 bp fragments by ion disruption. The first strand cDNA was synthesized with 6 base random primers and reverse transcriptase using RNA as template, and the second strand cDNA was synthesized with the first strand cDNA as template. The quality of the library was tested by Agilent 2100 Bioanalyzer, and the total concentration and effective concentration of the library were detected. After RNA extraction, purification, and library construction, these libraries were sequenced by Next-Generation Sequencing (NGS) based on Illumina NovaSeq 6000 sequencing platform (Hu Z. et al., 2020). Raw sequencing data were submitted to the National Center for Biotechnology Information (NCBI) Sequencing Read Archive (BioProject: PRJNA721900). The Reviewer link: https://dataview.ncbi.nlm.nih.gov/object/PRJNA721900?reviewer=57s3mhf1818q7pgoflsp2j06i6.

Reads containing adapters or ploy-N and low quality reads of raw data were removed to obtain clean data. Then, we calculated the Q20, Q30, and GC content of the cleaned data, which was stored in the FASTQ file format. After reading the filter, we used Trinity to clear the data for *de novo* assembly (Grabherr et al., 2011). Gene family clustering was then performed using the TIGR Gene Indices Clustering Tool (TGICL; version v2.0.6) to establish the final Unigenes (Ma et al., 2019). Finally, Unigene was used for subsequent Gene Ontology (GO^1^), Kyoto Encyclopedia of Genes and Genomes (KEGG^2^), Evolutionary Genealogy of Genes: Non-supervised Orthologous Groups (eggNOG^3^), SwissProt, Pfam annotation, ORF prediction, SSR prediction, etc. At the same time, the filtered sequence was compared to Unigene, and the Reads Count number of each Unigene was obtained. On this basis, the samples were further analyzed for expression difference analysis and enrichment analysis (Xie et al., 2018; Ma et al., 2019).

### Differentially Expressed Genes and Enrichment Analyses

DESeq (version 1.18.0) was used to analyze the difference of gene expression (Ding et al., 2020). The conditions for screening differentially expressed genes (DEGs) were as follows: | log2FoldChange| > 1, *P*-value < 0.05 (Wang et al., 2010). Principal component analysis (PCA) of each sample was implemented on the genes significantly expressed. GO and KEGG pathway enrichment analyses for DEGs were conducted with GO: Blast2go (Conesa and Gotz, 2008; Gotz et al., 2008; Camacho et al., 2009) and KEGG: KOBAS (Conesa and Gotz, 2008; Xie et al., 2011), respectively.

### Quantitative Reverse Transcriptase Polymerase Chain Reaction Analyses

To validate the RNA-seq results, three DEG genes were selected randomly for quantitative reverse transcriptase polymerase chain reaction (qRT-PCR) in CR vs. ZCR and LT vs. ZLT, respectively. The three DEG genes were TRINITY_DN11652_c0_g1, TRINITY_DN18023_c0_g2, and TRINITY_DN9657_c0_g1 in CR vs. ZCR. The three DEG genes were TRINITY_DN22630_c0_g1, TRINITY_DN15858_c0_g1, and TRINITY_DN30614_c0_g1 in LT vs. ZLT. qRT-PCR validation analyses were performed according to He et al. (2020), using Xs-Actin as the reference gene. The qRT-PCR experiments were performed on three biological replicates. Gene sequence, primer design, and actin gene are shown in Supplementary Material 1.

### Statistical Analysis

Statistical analysis was performed with the SPSS 22.0 software (IBM, United States) and Origin 9.0 (OriginLab, United States). One-way analysis of variances (ANOVA) with LSD tests was conducted to test significance difference between CR, LT, ZCR, and ZLT with the variation of soil heavy metal content in total biomass, vegetative and reproductive growth time, biomass ratio, Cd concentrations and accumulation of different plant parts, Cd accumulation ratio of different plant parts, the TF and BCF. The biomass of leave, Cd concentrations in litter and normal leaves and qRT-PCR validation of DEGs were determined by *T*-test at *P* < 0.05 or *P* < 0.01.

## Results

### Plant Growth Characteristics

**FIGURE 1:**
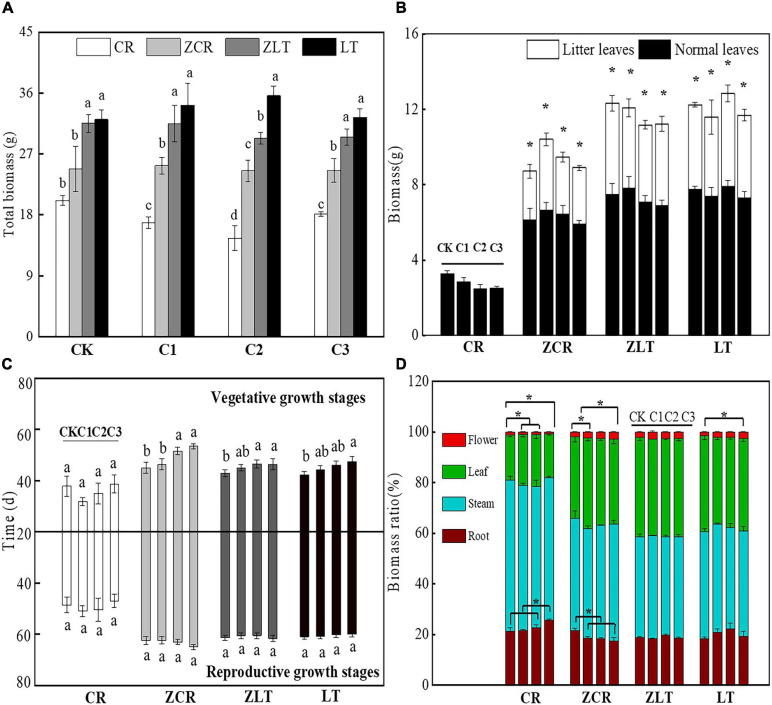
The growth characteristics of CR, ZCR, ZLT, and LT. **(A)** The total biomass of CR, ZCR, ZLT, and LT. Different small letters indicate significant differences among CR, ZCR, ZLT, and LT under the same Cd treatment [*P* < 0.05, degree of freedom (*df*): between groups = 3, within groups = 20]. **(B)** The biomass in litter and normal leaves of CR, ZCR, ZLT, and LT. “*” indicate significant differences between litter and normal leaves of CR, ZCR, ZLT, and LT (*P* < 0.05, *df*: between groups = 1, within groups = 10). **(C)** The vegetative and reproductive growth time of CR, ZCR, ZLT, and LT (*df*: between groups = 3, within groups = 20). **(D)** The biomass ratio of CR, ZCR, ZLT, and LT. “*” indicate significant differences in the same plants under different Cd treatment (*P* < 0.05, *df*: between groups = 3, within groups = 20).

Under C1–C3 treatment, the biomass of ZCR increased by 33.42, 40.52, and 26.24%, respectively, compared with that in CR (Figure 1A). As the soil Cd concentration increased, the reproductive allocation of ZCR increased from 2.06 to 3.17%, and the root biomass ratio decreased from 24.00 to 19.70%, which was opposite to that of CR (Figure 1D). Moreover, the average vegetative and reproductive growth durations of ZCR were 49.05 and 63.23 days, respectively, which were higher than 35.82 and 49.28 days of CR (Figure 1C). Interestingly, ZCR produced a large number of litter leaves throughout the growth period, consistent with LT and ZLT (Figure 1B). Surprisingly, the growth characteristics of ZLT were not significantly affected by hybridization (Figure 1). The data showed that hybridization changed the growth characteristics of CR and improved its tolerance to Cd.

### Cd Concentration and Accumulation in Plant

The Cd concentrations in different plant parts (roots, stems, and leaves) increased with increasing soil Cd levels (Table 1). Under C2 and C3 treatments, the Cd concentration and accumulation in leaves of ZCR were significantly higher than those of CR, while the Cd concentration of root was opposite (*P* < 0.05, Table 1). Except for the stem cadmium concentration and accumulation of LT under C3 treatment were higher than those of ZLT (Table 1), there was no significant difference in other treatments. Hybridization improves the Cd uptake and transportation of CR to Cd compared LT.

**TABLE 1:** The Cd concentrations and accumulation of different plant parts (root, stem, and leaf).

Treatment	Species	Cd concentration (mg/kg)			Cd accumulation (μ g/plant)		
		Root	Steam	Leaf	Root	Steam	Leaf
CK	CR	0.75 ± 0.06 d	0.95 ± 0.21 a	0.77 ± 0.21 c	2.63 ± 0.18 c	8.68 ± 0.93 a	0.23 ± 0.06 c
	ZCR	2.58 ± 0.09 b	0.74 ± 0.10 a	0.22 ± 0.07 b	15.86 ± 1.81 a	10.48 ± 3.43 a	2.00 ± 0.65 b
	ZLT	2.95 ± 0.06 a	0.56 ± 0.03 a	0.36 ± 0.03 a	18.52 ± 0.18 a	8.11 ± 0.88 a	4.81 ± 0.34 a
	LT	1.34 ± 0.12 c	0.65 ± 0.09 a	0.10 ± 0.02 bc	8.47 ± 0.22 b	10.00 ± 0.66 a	1.31 ± 0.24 b
C1	CR	16.43 ± 0.18 b	2.35 ± 0.21 a	1.17 ± 0.22 a	57.62 ± 5.62 b	22.72 ± 2.37 a	4.26 ± 1.11 b
	ZCR	22.46 ± 1.60 a	1.49 ± 0.12 b	1.53 ± 0.23 a	120.38 ± 11.43 a	18.29 ± 2.99 a	15.88±3.13 a
	ZLT	9.41 ± 0.96 c	1.14 ± 0.07 b	1.63 ± 0.26 a	59.26 ± 9.58 b	16.06 ± 1.96 a	20.68 ± 2.53 a
	LT	6.22 ± 0.69 c	1.15 ± 0.09 b	1.37 ± 0.11 a	51.95 ± 0.27 b	20.48 ± 2.21 a	19.72 ± 1.80 a
C2	CR	47.84 ± 3.97 a	3.84 ± 0.82 a	1.86 ± 0.42 b	180.61 ± 13.84 a	32.92 ± 3.65 ab	5.73 ± 1.15 b
	ZCR	35.02 ± 1.70 b	2.73 ± 0.23 a	2.90 ± 0.02 a	190.46 ± 3.91 a	36.30 ± 4.25 a	29.60 ± 2.42 a
	ZLT	18.99 ± 1.72 c	1.47 ± 0.10 b	2.39 ± 0.16 ab	110.29 ± 5.16 b	17.82 ± 0.42 c	27.91 ± 1.84 a
	LT	14.26 ± 0.68 c	1.56 ± 0.22 b	2.40 ± 0.02 ab	104.42 ± 3.41 b	24.37 ± 3.62 bc	32.35 ± 1.22 a
C3	CR	60.32 ± 2.58 a	4.71 ± 0.21 b	3.70 ± 0.89 b	289.04 ± 16.42 a	47.60 ± 2.47 c	12.38 ± 2.00 b
	ZCR	49.68 ± 3.02 b	5.65 ± 0.86 ab	8.63 ± 0.82 a	286.63 ± 26.50 a	79.12 ± 11.61 b	81.05 ± 7.40 a
	ZLT	28.22 ± 2.05 c	4.81 ± 0.30 b	8.23 ± 0.74 a	158.56 ± 14.30 b	62.44 ± 5.78 bc	104.03 ± 10.44 a
	LT	29.11 ± 5.97 c	7.15 ± 0.46 a	8.67 ± 2.18 a	241.83 ± 43.44 ab	105.18 ± 6.70 a	103.40 ± 27.16 a

*Different small letters indicate significant differences among CR, ZCR, ZLT, and LT at the same treatment (*P* < 0.05, *df*: between groups = 3, within groups = 20).*

**FIGURE 2:**
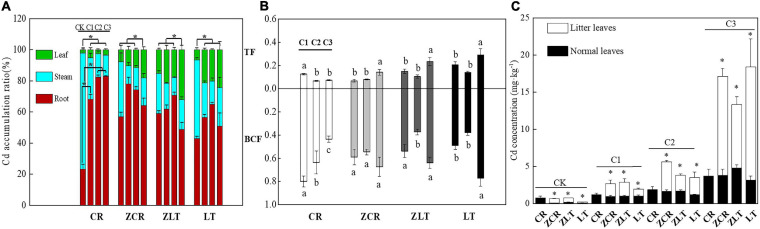
Transport and distribution of cadmium in plants. **(A)** The Cd accumulation ratio of different plant parts (root, stem, leaf) (*df*: between groups = 3, within groups = 20). **(B)** The TF and BCF of CR, ZCR, ZLT, and LT. Different small letters and “^∗^” indicate significant differences in the same species under different Cd treatment (*P* < 0.05, *df*: between groups = 2, within groups = 15). **(C)** The Cd concentration of litter and normal leaves in CR, ZCR, ZLT, and LT. “^∗^” indicate significant differences between litters and normal leaves of CR, ZCR, ZLT, and LT (*P* < 0.05, *df*: between groups = 1, within groups = 10).

**FIGURE 3:**
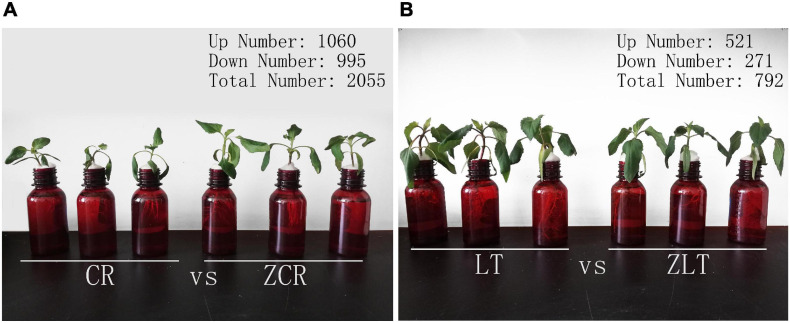
Statistics for differentially expressed genes. **(A)** DEGs of ZCR under Cd stress. **(B)** DEGs of ZLT under Cd stress.

Under C1–C3 treatment, the Cd accumulation ratio in root of ZCR decreased from 77.89 to 64.15%, and the Cd accumulation ratio of leaf increased from 7.84 to 18.14%. On the contrary, the Cd accumulation ratio in root of CR increased from 68.10 to 82.81%, and the Cd accumulation ratio of leaf decreased from 5.04 to 3.55% (Figure 2A). The BCF and TF values of CR decreased with increasing soil Cd levels, while ZCR was opposite (Figure 3B). In other words, the increased Cd tolerance in ZCR is not caused by a decreased Cd uptake and transportation. Interestingly, Cd in plant leaves was redistributed, the Cd concentrations in litter leaves of ZCR, ZLT, and LT were almost three times as high as that in normal leaves (Figure 2C).

### Differentially Expressed Genes Analysis

The basic results of transcriptome sequencing of CR, ZCR, ZLT, and LT can be found in Supplementary Tables 1, 2; For GO annotations and KEGG pathway analysis, see Supplementary Figures 5, 6. Hybridization is a common gene exchange in nature. It involves in the recombination of a large number of genes on intact chromosomes, which can change certain biological traits. Under Cd stress, the gene transcription level of hybrid may be different from that of parents, which may lead to differences in growth and Cd accumulation between hybrid and parents. A total of 2055 (1060 up and 995 down) DEGs were detected in the leaves of Cd-stressed ZCR compared with that of CR (CR vs. ZCR, Figure 3A), while only 792 (521 up and 271 down) were detected in ZLT compared with LT (LT vs. ZLT, Figure 3B). Detailed data on DEGs of ZCR and ZLT are available in Supplementary Materials 2, 3, respectively. In addition, the number of DEGs in CR vs. ZLT and LT vs. ZCR were 14,625 (7780 up and 6917 down) and 20,295 (10,522 up and 9773 down), respectively, indicating that the hybrid was more similar to the female parent in gene expression (Supplementary Figure 7).

**FIGURE 4:**
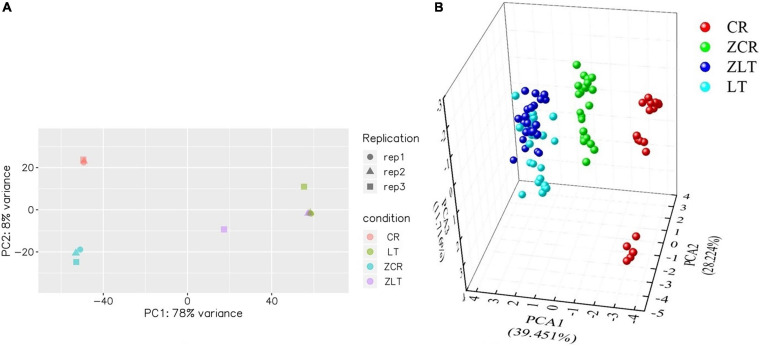
Principal component analysis analysis of DEGs expression and pot experiment data. **(A)** PCA analysis of DEGs expression of CR, ZCR, ZLT, and LT. **(B)** PCA analysis of pot experiment based on the physiological parameters of CR, ZCR, ZLT, and LT treated with CK, C1, C2, C3.

Under cadmium stress, the number of DEGs in ZCR was more than twice that in ZLT (Figure 3). PCA analysis of DEGs expression showed that CR and ZCR had significant differences, while LT and ZLT had certain similarities (Figure 4A). Moreover, the PCA results of pot experiment data (Figure 4B) and DEGs expression (Figure 4A) showed surprising consistency. We speculated that hybridization had a greater impact on CR than LT under cadmium stress, which led to different performance between CR and ZCR (Supplementary Figure 8).

### GO and KEGG Pathway Enrichment Analyses of DEGs

**FIGURE 5:**
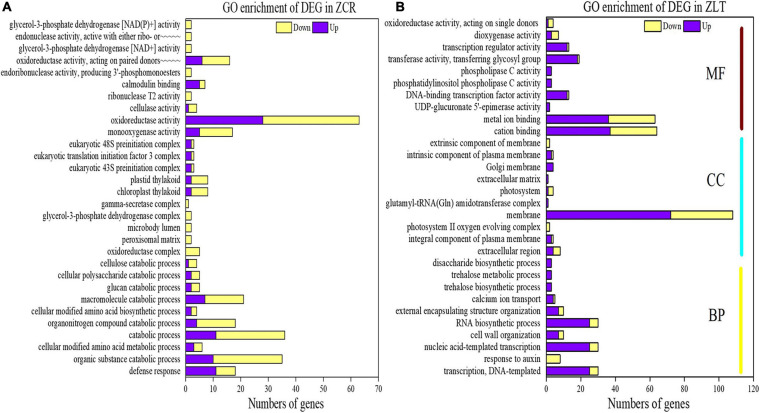
Gene Ontology enrichment of DEGs. **(A)** GO enrichment of DEGs in ZCR. **(B)** GO enrichment of DEGs in ZLT. In panel **(A)**, the full name of “endonuclease activity, active with either ribo- or∼∼∼∼∼” is “endonuclease activity, active with either ribo- or deoxyribonucleic acids and producing 3′-phosphomonoesters”; the full name of “oxidoreductase activity, acting on paired donors∼∼∼∼∼” is “oxidoreductase activity, acting on paired donors, with incorporation or reduction of molecular oxygen.”

In order to further explore the molecular mechanism of hybridization changing CR and LT, GO and KEGG pathway enrichment analysis was performed on the differential genes of ZCR and ZLT. We selected the top 10 GO terms for each GO category for presentation. GO analysis of DEGs in ZCR showed that in the biological process category, DEGs were mainly enriched in defense response, organic substance catabolic process, catabolic process, organonitrogen compound catabolic process, and macromolecule catabolic process; In the cell component category, DEGs were mainly enriched in the chloroplast thylakoid and plastid thylakoid; In the molecular functional categories, DEGs were mainly enriched in monooxygenase activity, oxidoreductase activity and “oxidoreductase activity, act on paired donors, with incorporation or reduction of molecular oxygen” (Figure 5A). GO analysis of DEGs in ZLT showed that in the biological process category, DEGs were mainly enriched in “transcription, DNA-templated,” nucleic acid-templated transcription and RNA biosynthetic process; In the cell component category, DEGs were mainly enriched in membrane; In the molecular functional categories, DEGs were mainly enriched in cation binding, metal ion binding, and transferase activity, transferring glycosyl groups (Figure 5B). The down-DEGs of ZCR accounted for 64.70%, while the up-DEGs of ZLT accounted for 68.98% (Figure 5), suggesting that under cadmium stress, hybridization gave different response strategies to invasive species LT and its native congener CR.

**FIGURE 6:**
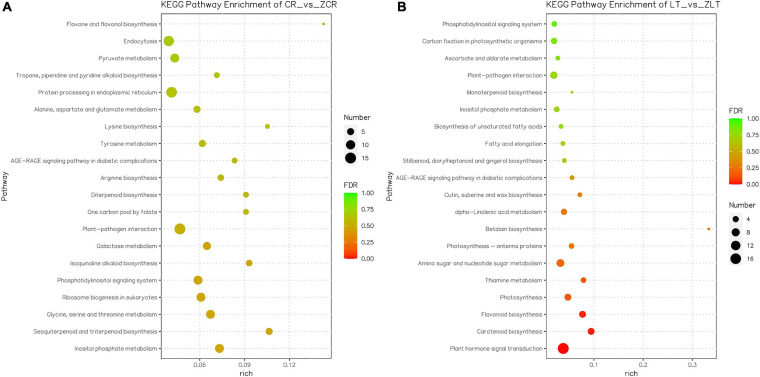
Kyoto Encyclopedia of Genes and Genomes enrichment of DEGs. **(A)** KEGG enrichment of DEGs in ZCR. **(B)** KEGG enrichment of DEGs in ZLT.

To identify the biological pathways triggered by Cd stress, all DEGs were assigned to the KEGG database for functional annotations, and further KEGG pathway enrichment analysis. A total of 381 out of 2055 DEGs in ZCR were enriched in 90 KEGG pathways, and 8 pathways were significantly enriched (Figure 6A, *P*-value < 0.05); while in ZLT, a total of 135 out of 792 DEGs were enriched in 63 KEGG pathways, and 10 pathways were significantly enriched (Figure 6B, *P*-value < 0.05). Only four KEGG enrichment pathways in ZCR and ZLT were the same. The uniquely enriched pathways indicate there exist different mechanisms in response to Cd stress (Figure 6). Since there are a large number of DEGs in CR vs. ZLT and LT vs. ZCR (Supplementary Figure 7), GO terms and KEGG pathways obtained by specific algorithms will be significantly different, which can also explain why GO (Supplementary Figure 9A) and KEGG analysis (Supplementary Figure 10A) results are different between CR vs. ZCR and CR vs. ZLT. Similar phenomena are also found in LT vs. ZLT and LT vs. ZCR (Supplementary Figures 9B, 10B).

### RNA-Seq Validation by qRT-PCR

**FIGURE 7:**
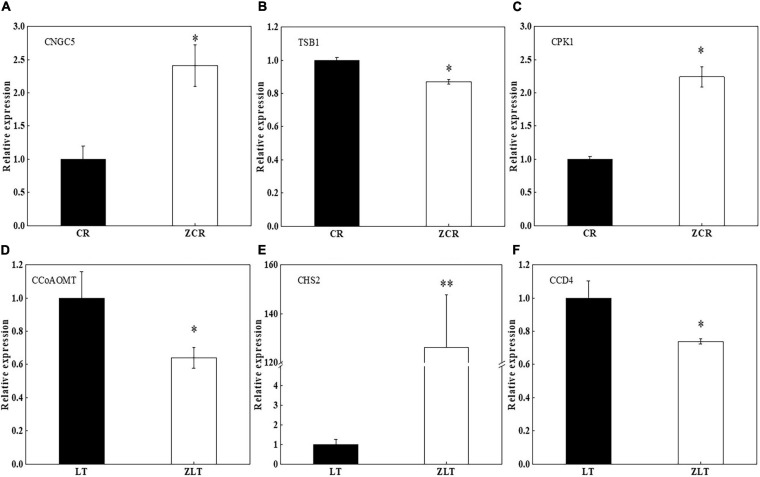
Quantitative reverse transcriptase polymerase chain reaction validation of DEGs. **(A–C)** Three DEG genes were selected randomly for qRT-PCR in CR vs ZCR. **(D–F)** Three DEG genes were selected randomly for qRT-PCR in LT vs ZLT. TSB1, tryptophan synthase beta chain 1; CNGC5, cyclic nucleotide-gated ion channel 5; CPK1, calcium-dependent protein kinase 1; CCoAOMT, caffeoyl-CoA *O*-methyltransferase; CHS2, chalcone synthase 2; CCD4, carotenoid cleavage dioxygenase 4. ^∗^*P* < 0.05; ^∗∗^*P* < 0.01. *df*: between groups = 1, within groups = 4.

Genes CNGC5 and CPK1 in leaves of ZCR showed significant higher expression level than that of CR (Figures 7A,C, *P* < 0.05). The expression of TSB1 in leaves of ZCR was lower than that of CR (Figure 7B, *P* < 0.05). Genes CCoAOMT and carotenoid cleavage dioxygenase 4 (CCD4) in leaves of ZLT showed significant lower expression level than that of LT (Figures 7D,F, *P* < 0.05). The expression of CHS2 in leaves of ZLT was higher than that of LT (Figure 7E, *P* < 0.01).

## Discussion

### Hybridization Improved the Tolerance of Native Congener CR to Cd

Usually, hybridization can increase the probability of successful invasion of exotic invasive plant. Exotic invasive plants often face new environmental pressures in entering a new environment and need to overcome many biological and abiotic barriers. Exotic invasive plants can adapt to new selection pressure in a short time through hybridization with native species (Blackburn et al., 2011). For native species, hybridization with invasive species broke the stable relationship between native species and other biological (plant, animal), abiotic factors (temperature, water) in the long history of coevolution, changed the local adaptability of native species, and showed a negative impact on native species (Verhoeven et al., 2011).

However, our results of the pot experiment showed that the biomass of ZCR was significantly higher than that of CR and did not significantly decrease with the increase of Cd stress, indicating that hybridization with LT significantly increased the tolerance of its native congener CR to Cd. When the concentration of heavy metals in the soil remains below the critical concentration which inhibits the growth and development of plants, plant biomass does usually not decrease. Once this threshold is exceeded, the growth and development of plants is inhibited, and following symptoms such as yellowing of leaves and reduction of plant height, this inhibition is reflected in a significant decrease in plant biomass (Liu et al., 2019). Concerning the question of how the plant deals with the damage caused by the increase of Cd content in the soil, and consequently in plant tissues, studies have shown that plants can enhance their ability to cope with stress by altering the distribution of photosynthetic products in the aboveground and belowground parts (Mealor and Hild, 2007; Quan et al., 2015). For example, at low nutrient levels, plants allocate more energy to the nutrient-absorbing organs, increasing the R/S (root to shoot ratio) and nutrient uptake. On the contrary, at high nutrient levels, plants allocate more energy to the carbon assimilation organs (leaves), enhancing photosynthesis. In this study, as the soil Cd concentration increased, CR increased root biomass and reduced reproductive allocation (Figure 1D and Supplementary Figure 11A), and accumulated a large amount of Cd in roots (Figures 2A,B). These results indicated that CR may decrease the damage caused by the accumulation of Cd in the roots by allocating the photosynthetic products to the root system in order to maintain its water and nutrient absorption, thereby reducing the concentration of Cd in aboveground parts, but this distribution pattern seriously affects the reproductive capacity of CR. Surprisingly, ZCR showed distinct biomass allocation and Cd accumulation strategies compared with CR. The data showed that ZCR not only reduced root biomass and increased reproductive biomass (Figure 1D and Supplementary Figure 11C), but also increased Cd content in leaves (Figure 2A). Theoretically, this is a self-contradictory allocation strategy. However, Cd redistribution in leaves of ZCR may play an important role. A large amount of senescent leaves was produced by ZCR (Figure 1B) and the Cd concentration of senescent leaves was three times as much as the green leaves (Figure 2C). Biological and abiotic stresses such as high temperature, drought, pathogens, and heavy metals often promote plant senescence. Plant senescence helps plants adapt to environmental changes and maintain efficient energy utilization, so that plants can also complete their life cycle under stress conditions (Xiao et al., 2014; Yang and Guo, 2014). For example, the Cd concentration of senescent leaves in *Schinus molle* L. was higher than young leaves (Pereira et al., 2017). The Cd and arsenic (As) concentration of senescent leaves in rice increased significantly compared young leaves (Shi G. L. et al., 2019). Plant senescence induced by abscisic acid (ABA) and SA contributes to the transportation of heavy metals, and Cd content in shoots of *Festuca arundinacea* S. increases significantly (Hza et al., 2020). We speculate that this is the adaptive strategy of ZCR for resisting Cd toxicity. A large amount of Cd is stored in senescent leaves and eliminated from the plant when the leaves fall off, which not only reduces the Cd content in the plant, but also reduces the toxicity of Cd to young and mature leaves (Sridhar et al., 2005; Wang and Su, 2005; Qin et al., 2013).

Reproductive events usually determine population and community dynamics in future generations, affecting evolutionary processes (Sherry et al., 2007). It is imperative to understand the impact of hybridization and Cd change on reproductive traits. The data showed that hybridization not only increased the reproductive allocation ratio of CR, but also prolongs the vegetative and reproductive time. Moreover, hybridization did not significantly reduce the photosynthetic rate of CR at vegetative and reproductive stages (Supplementary Figure 12), which means that ZCR can produce more photosynthetic products, improve its survival probability and the population size (Parepa et al., 2013; He and Ma, 2018). Relevant research has proved that invasive plants can interfere with pollination of native plants in invasive sites (Brown et al., 2002; Kandori et al., 2009; Flanagan et al., 2010; Sun et al., 2018). The existence of hybrids between CR and LT in nature indicates that pollination of CR had been disturbed. In this study, the number of flowers and reproductive efficiency index of ZCR was significantly higher than those of CR under all treatment (*P* < 0.05, Supplementary Figure 13). The high reproductive allocation of hybrids may enhance this interference, which will further inhibit the reproductive process of CR and prevent it from producing enough seeds to maintain population renewal. From 2015 to the present, our research team had been conducting field plant population surveys in Shenyang, Fuxin, Chaoyang, and Jinzhou, Liaoning Province, China. The survey results showed that in the mixed community of CR and LT, the population number of CR continued to decrease, and LT and hybrids became the dominant populations. Although there is currently no direct evidence showing that hybridization with LT affects the population of CR, we should consider the potential threat of hybridization to CR population extinction (Wu et al., 2013; Ni et al., 2014).

### The Role of DEGs of ZCR and ZLT in Response to Cd Stress

Kyoto Encyclopedia of Genes and Genomes analysis revealed that many DEGs of ZCR and ZLT were associated with several pathways, particularly in Glycine, serine, and threonine metabolism, Plant–pathogen interaction, Carotenoid biosynthesis, Flavonoid biosynthesis (Figure 4). The DEGs of ZLT were involved in ABA synthesis and decomposition. The gene expression encoding CCD4 (TRINITY_DN 22630_c0_g1), phytoene synthase 2 (PSY2, TRINITY_DN20908_c0_g1), and 9-cis-epox-ycarotenoid dioxygenase (NCED, TRINITY_ DN489_c0_g1, TRINITY_DN24283_c0_g1) in ZLT were down-regulated, while the genes encoding abscisic acid 8′-hydroxylase 2-like (ABA8ox2-like, TRINITY_DN1248_c0_g1) and abscisic acid receptor (PYL4, TRINITY_DN26835_c1_g1) were up-regulated. The DEGs of ZCR were involved in the regulation of ABA induction. The gene expression encoding cyclic nucleotide-gated ion channel (CNGC, TRINITY_ DN4064_c0_g1, TRINITY_DN11652_c0_g1), calcium-dependent protein kinase (CPK, TRINITY_DN10060_c0_g1, TRINITY_DN18023_c0_g2), WRKY transcription factor 33 (WRKY33, TRINITY_DN72884_c1_g 1), calmodulin-like protein 3 (CML3, TRINITY_DN1385_c0_g1), and PTI1-like tyrosine-protein kinase 3 (PTI3, TRINITY_DN23545_c0_g2) in ZCR were up-regulated, while the genes encoding respiratory burst oxidase homolog protein C (RBOHC, TRINITY_ DN74133_c-0_g1) was down-regulated. In this study, the hybrids enhanced the transportation of Cd from root to shoot with the increase of cadmium stress (Figures 2A,B). Moreover, a large number of senescent leaves were produced in hybrids (Figure 1B), and the concentration of Cd in the senescent leaves was almost three times that in the young and mature leaves (Figure 2C). These imply a important role of ABA in hybrids response to Cd stress.

Abscisic acid is one of the most important plant hormones, and it plays vital roles in plant growth, development, seed germination, embryo morphogenesis, stomatal closure, fruit ripening, leaf senescence and responses to biotic and abiotic stress (Zhang W. et al., 2019; Feng et al., 2020; Pan et al., 2020). Plant endogenous ABA content and gene expression were affected by heavy metal stress, and ABA content was positively correlated with plant tolerance to heavy metals (Shi W. G. et al., 2019; Hu B. et al., 2020). Studies on the response of plants such as *Arabidopsis*, *Solanum tuberosum*, *Sedum alfredii*, *Malus hupehensis* to heavy metal Cd have shown that ABA plays a positive role in alleviating the accumulation and toxicity of heavy metals and metalloid compounds (Stroiński et al., 2013; Sheng et al., 2018; Han et al., 2019; Tao et al., 2019; Zhang W. et al., 2019). Three major pathways involved in the detoxification of toxic metals can be triggered by ABA, inhibiting the uptake (Zhang L. et al., 2019; Pan et al., 2020), altering the translocation from root to shoot (Pompeu et al., 2017; Shi W. G. et al., 2019; Hza et al., 2020), and promoting the conjugation with chelators (Lu et al., 2020). Hsu and Kao (2008) found that regulation of endogenous ABA biosynthesis can reduce Cd uptake by rice seedlings (Hsu and Kao, 2008). Hu B. et al. (2020) speculated that ABA may inhibit As (V) uptake by Arabidopsis thaliana through WRKY6-PHT1, 1 pathway (Hu B. et al., 2020). ABA can also regulate plant stomata through phosphorylation of guard cell membrane localization transporters mediated by kinases such as calcium-dependent kinases (CDPKs), which may prevent the transport of metal ions and quasimetals to buds (Pornsiriwong et al., 2017; Hu B. et al., 2020). The synthesis of ABA in higher plants is dominated by carotenoid C40 pathway (Zhang et al., 2009). ABA synthesis requires the involvement of PSY, CCD, NCED, and other related enzymes (Leng et al., 2014), and NCED is considered to be a key rate-limiting enzyme in ABA synthesis pathway (Sun et al., 2012). Metabolic decomposition of ABA mainly includes hydroxylation of abscisic acid 8′-hydroxylase (CYP707A) and glycosylation of glucosyltransferase (AOG/GT) (Zhang et al., 2009).

Cyclic nucleotide-gated ion channels, CPK, CML, PTI, and WRKY are induced and regulated by ABA (Hu B. et al., 2020). CNGCs are involved in the regulation of Ca^2+^ influx in plants. Moreover, CNGCs, as non-selective ion channels, are likely to be the channels for heavy metal ions to enter cells (Sunkar et al., 2000; Guo et al., 2010). The CNGC channel (NtCBP4, Calmodu-lin Binding Protein 4) in *Nicotiana tabacum* was proved to be the pathway for heavy metal ions to enter cells through plasma membrane (Arazi et al., 1999). The several members of the *Arabidopsis* CNGC family have recorded potential roles in plant tolerance to heavy metal and uptake of Pb^2+^ and Cd^2+^, like AtCNGC11/15/19 (Moon et al., 2019). ABA can induce the expression of CNGC1.1/1.2 and enhance the absorption and transportation of Pb^2+^ by *Populus* × *Canescens* (Shi W. G. et al., 2019). The influx of Ca^2+^ activates CML to inhibit the activity of CNGCs and prevents the intracellular calcium concentration from soaring. In addition, the influx of Ca^2+^ combines with CML to participate in the downstream hypersensitive response (HR) or autoimmune cascade reaction (Liu et al., 2015). CPK can regulate the ion channels of guard cells, thereby regulating stomatal movement in plant leaves (Yip and Boudsocq, 2019). CPK can also regulate ROS production by phosphorylation of respiratory burst oxidase homologue D (RBOHD; Boudsocq et al., 2010). WRKY transcription factor is a unique transcription factor in plants, which is involved in the expression of senescence-related genes and can be highly induced by ABA (Rushton et al., 2010). WRKY can act as a positive and negative regulator of plant development and defense. WRKY12 (Han et al., 2019) and WRKY13 (Sheng et al., 2018) negatively regulate Cd accumulation and tolerance in *Arabidopsis*. Overexpression of WRKY12 and WRKY13 reduces Cd accumulation and enhances Cd tolerance. The overexpression of GmWRKY142 in *Arabidopsis* and soybean hairy roots reduced Cd uptake and significantly enhanced tolerance (Zhang C. Z. et al., 2020). However, overexpression of CaWRKY41 in *Arabidopsis* could enhance the absorption of Cd and Zn and reduce tolerance (Dang et al., 2019). Except regulating the absorption and tolerance of heavy metals, WRKY also plays an important role in leaf senescence, AtWRKY54 and AtWRKY70 negatively regulate leaf senescence in *Arabidopsis* (Besseau et al., 2012). OsWRKY42 overexpression lines in rice showed early senescence, increased hydrogen peroxide content of reactive oxygen species and decreased chlorophyll content (Han et al., 2014).

We speculated the up-regulated expression of CNGC5/20 by ABA in ZCR enhanced Cd absorption and transportation, and the down-regulation of RBOHC expression reduced oxidative stress. Moreover, ABA-induced up-regulated expression of WRKY33 regulates Cd transport and accumulation in senescent and normal leaves, which may contribute to protect young and mature leaves. The expression levels of four ABA-synthesis genes, CCD4, PSY2, NCED1, and NCED2 were found to be lower in ZLT than those of LT, and the expression level of ABA hydroxylase genes CYP707 was higher in ZLT than in LT. Differential expression of these genes may contribute to reduce Cd accumulation in young and mature leaves in ZLT. ZCR showed some similarities with LT and ZLT in growth characteristics, Cd accumulation and distribution characteristics (Figure 3), but the internal regulatory mechanisms were still different. We speculated that ABA played vital role in Cd transportation of hybrids and Cd distribution in senescent and normal leaves (Pereira et al., 2017; Shi W. G. et al., 2019; Hza et al., 2020). The relationship among the ABA synthesis and signal transduction, Cd absorption, and transportation, and plant senescence requires further investigation to reveal detailed mechanisms involved in Cd distribution and accumulation in plants.

## Conclusion

The interaction between biological invasion and environmental pollution should be carefully studied. In this study, the hybridization with an invasive plant of LT improves the tolerance of CR to Cd, and ZCR showed completely different Cd response strategies compared with CR. Hybridization with an invasive plant of LT may enhance the survival competitive pressure and the extinction risk of CR. Therefore, the impact of plant invasion and environmental pollution on native plant is worthy of our attention.

## Data Availability Statement

The original contributions presented in the study are publicly available. This data can be found here: Raw sequencing data were submitted to the National Center for Biotechnology Information (NCBI) Sequencing Read Archive (BioProject: PRJNA721900).

## Author Contributions

BQ, PT, and CX conceived the study design. YG and CG was responsible for the sample preparation and experimentation. CX, WC, and GZ performed the bioinformatic analysis. CX completed the initial manuscript. BQ and PT revised the manuscript. All the authors read and approved the final version of the manuscript.

## Conflict of Interest

The authors declare that the research was conducted in the absence of any commercial or financial relationships that could be construed as a potential conflict of interest. The reviewer MFI declared a shared affiliation, with no collaboration, with several of the authors, CX, YG, and BQ, to the handling editor at the time of the review.

## Publisher’s Note

All claims expressed in this article are solely those of the authors and do not necessarily represent those of their affiliated organizations, or those of the publisher, the editors and the reviewers. Any product that may be evaluated in this article, or claim that may be made by its manufacturer, is not guaranteed or endorsed by the publisher.

## Footnotes

1
http://geneontology.org/

2
http://www.kegg.jp/

3
http://eggnog.embl.de/version_3.0/

## Supplementary Material

Supplementary Figure 1 Sampling point map of parent materials.

Supplementary Figure 2 Schematic diagram of LT and CR seed acquisition.

Supplementary Figure 3 Schematic diagram of hybrid seed acquisition.

Supplementary Figure 4 The seeds of *Xanthium strumarium* (LT) and *Xanthium sibiricum* (CR).

Supplementary Figure 5 The GO classification of the annotated genes.

Supplementary Figure 6 The KEGG Pathway of the annotated genes.

Supplementary Figure 7 Volcano map of DEGs. **(A)** Volcano map of CR vs. ZLT. **(B)** Volcano map of LT vs. ZCR.

Supplementary Figure 8 Image schema of PCA analysis of DEGs expression and pot experiment data.

Supplementary Figure 9 Gene Ontology enrichment of DEGs. **(A)** GO enrichment of CR vs. ZLT. **(B)** GO enrichment of LT vs. ZCR.

Supplementary Figure 10 Kyoto Encyclopedia of Genes and Genomes enrichment of DEGs. **(A)** KEGG enrichment of CR vs. ZLT. **(B)** KEGG enrichment of LT vs. ZCR.

Supplementary Figure 11 Biomass ratio and Soil Cd treatment-response relationships of CR, ZCR, ZLT, and LT. RR, root biomass ratio; SR, stem biomass ratio; LR, leave biomass ratio; RA, reproductive allocation.

Supplementary Figure 12 *P*_*max*_ of CR, ZCR, ZLT, and LT at different growth stages. **(A)** *P*_*max*_ of CR, ZCR, ZLT, and LT at vegetative growth stages. **(B)** *P*_*max*_ of CR, ZCR, ZLT, and LT at reproductive growth stages. Within the same Cd concentration, different small letters indicate significant differences in the same plants (*P* < 0.05; *df*: between groups = 3, within groups = 20).

Supplementary Figure 13 The number of flower and reproductive efficiency index of CR, ZCR, ZLT, and LT under different Cd treatment. **(A)** The number of flower in CR, ZCR, ZLT, and LT. **(B)** The reproductive efficiency index of CR, ZCR, ZLT, and LT. Different small letters indicate significant differences among CR, ZCR, ZLT, and LT at the same treatment (*P* < 0.05; *df*: between groups = 3, within groups = 20).

Supplementary Table 1 Summary statistics of raw reads and clean reads.

Supplementary Table 2 Overall statistics of transcript and Unigene sequences.
