# Supplementary material for: Hybridization With an Invasive Plant of Xanthium strumarium Improves the Tolerance of Its Native Congener X. sibiricum to Cadmium
Source: Front Plant Sci. 2021 Jul 29;12:696687. doi: 10.3389/fpls.2021.696687 (PMC8358311; doi:10.3389/fpls.2021.696687)
Supplement: Supplementary Figure 1 — Sampling point map of parent materials. [file Data_Sheet_1.doc]

Supplementary data


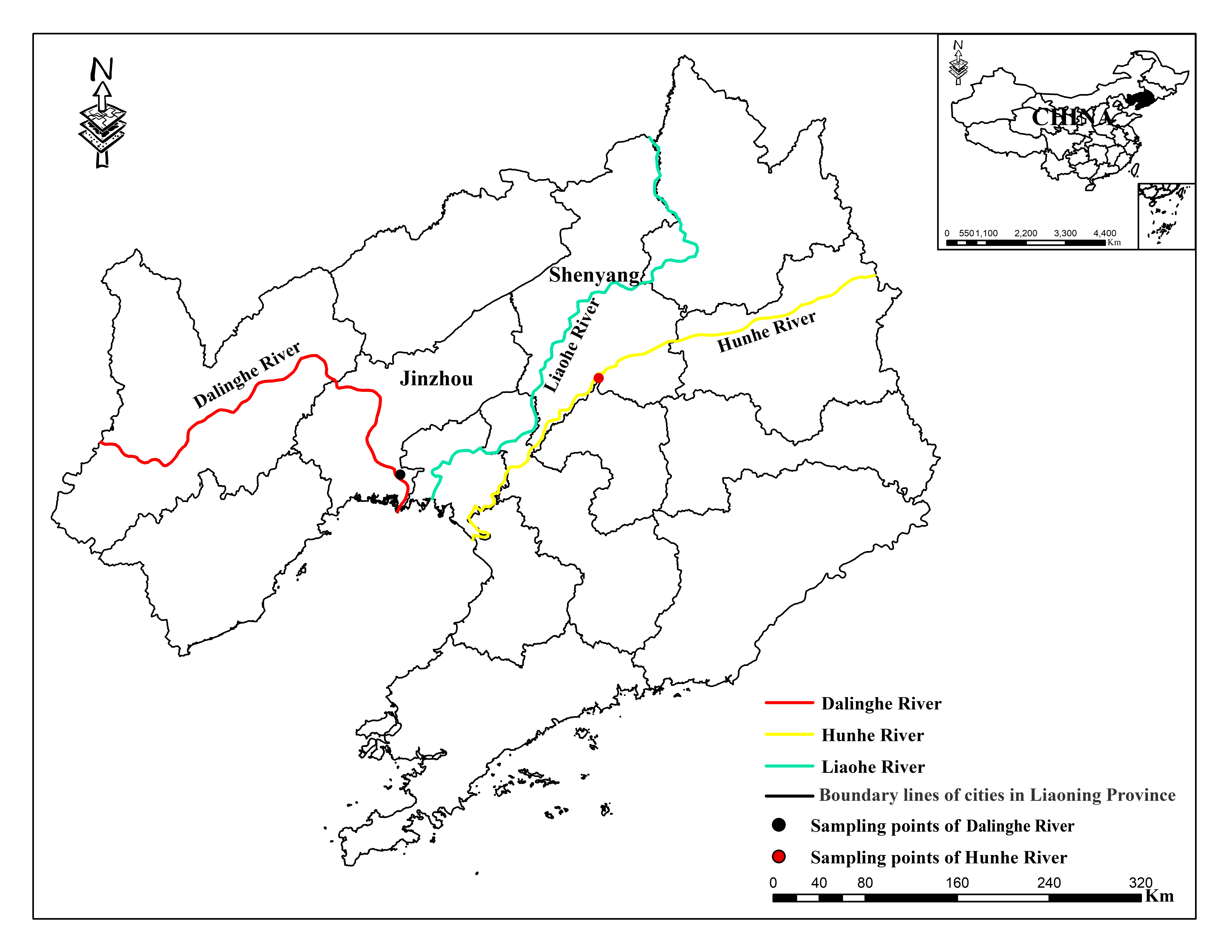


Supplementary Figure 1 Sampling point map of parent materials.


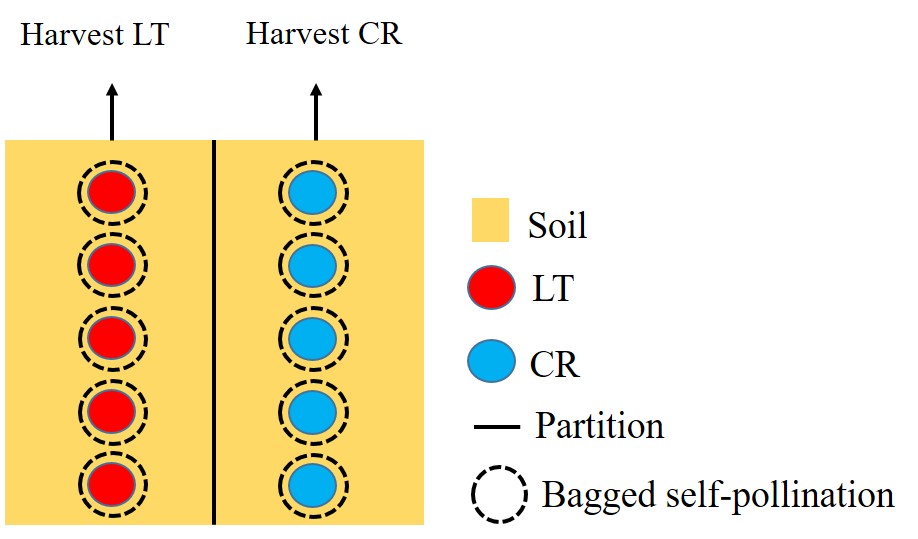


Supplementary Figure 2 Schematic diagram of LT and CR seed acquisition.


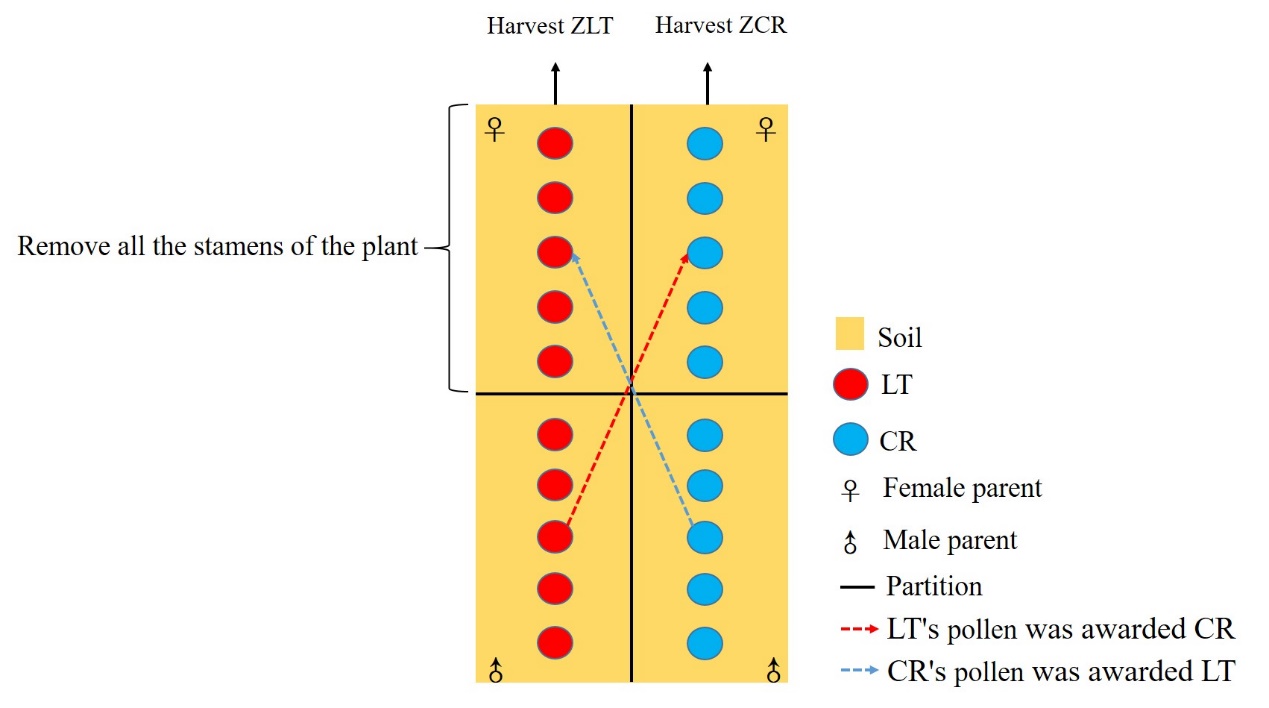


Supplementary Figure 3 Schematic diagram of hybrid seed acquisition.


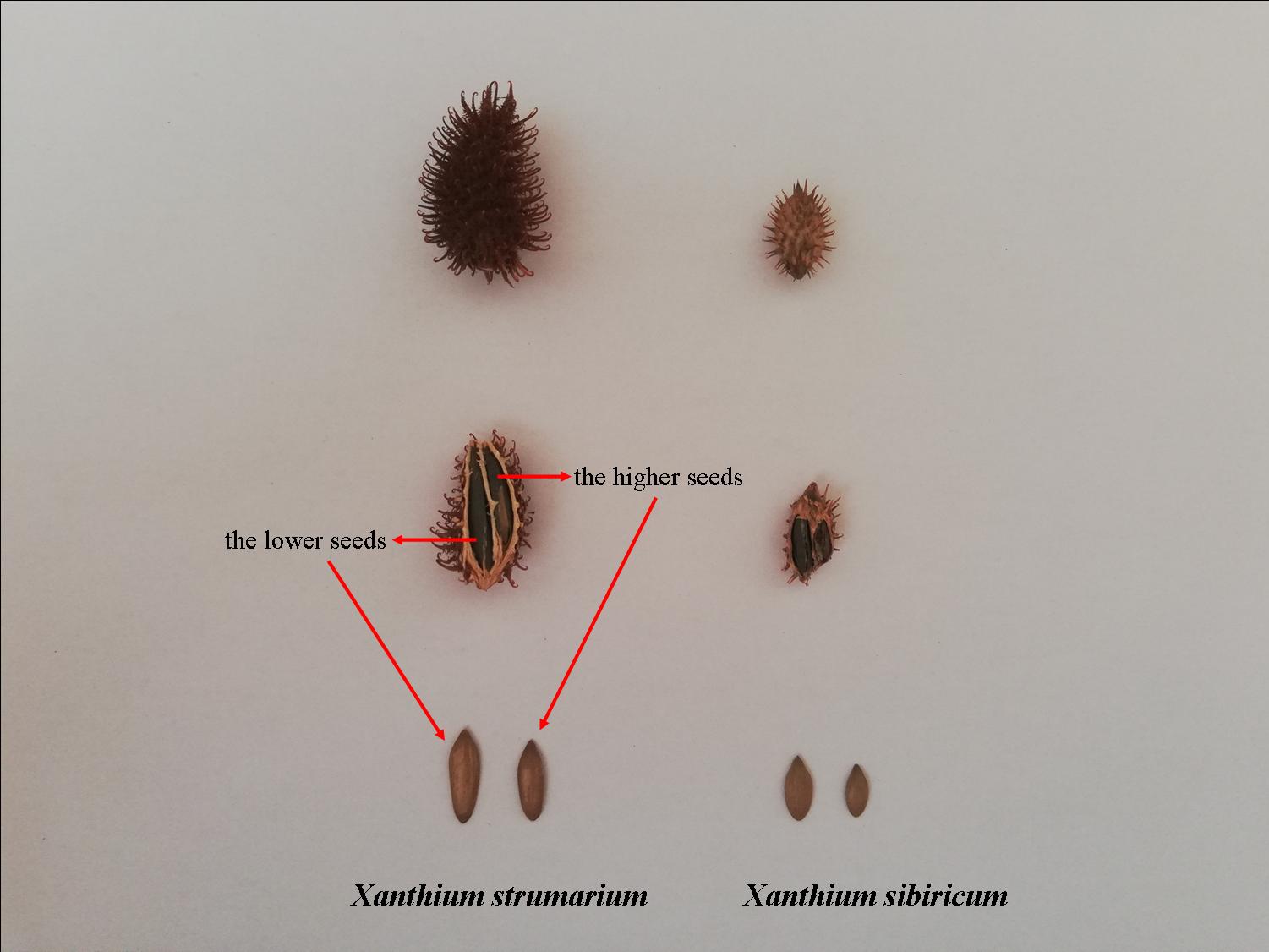


Supplementary Figure 4 The seeds of *Xanthium strumarium* (LT) and *Xanthium sibiricum* (CR).

Supplementary Table 1 Summary statistics of raw reads and clean reads.

| Sample | Reads No. | Bases (bp) | Q30 (bp) | N (%) | Q20 (%) | Q30 (%) | Clean Reads % |
| --- | --- | --- | --- | --- | --- | --- | --- |
| CR1 | 46883654 | 7032548100 | 6572782289 | 0.000976 | 97.66 | 93.46 | 92.96 |
| CR2 | 41405562 | 6210834300 | 5777763169 | 0.000985 | 97.48 | 93.02 | 93.46 |
| CR3 | 42720870 | 6408130500 | 5990189164 | 0.000978 | 97.68 | 93.47 | 93.5 |
| LT1 | 39651054 | 5947658100 | 5559011616 | 0.000973 | 97.66 | 93.46 | 93.97 |
| LT2 | 39348064 | 5902209600 | 5491676188 | 0.00099 | 97.5 | 93.04 | 91.08 |
| LT3 | 40459044 | 6068856600 | 5655904479 | 0.000985 | 97.54 | 93.19 | 92.8 |
| ZCR1 | 42419302 | 6362895300 | 5941691802 | 0.000976 | 97.62 | 93.38 | 91.78 |
| ZCR2 | 42673330 | 6400999500 | 5978775013 | 0.00098 | 97.65 | 93.4 | 92.79 |
| ZCR3 | 41931132 | 6289669800 | 5851541150 | 0.000985 | 97.49 | 93.03 | 93.22 |
| ZLT1 | 41802750 | 6270412500 | 5844242859 | 0.000985 | 97.57 | 93.2 | 91.67 |
| ZLT2 | 42250072 | 6337510800 | 5918062600 | 0.00098 | 97.67 | 93.38 | 92.44 |
| ZLT3 | 45314304 | 6797145600 | 6342845373 | 0.00097 | 97.6 | 93.31 | 90.75 |

Supplementary Table 2 Overall statistics of transcript and Unigene sequences.

|  | Transcript | Unigene |
| --- | --- | --- |
| Total Length (bp) | 315689403 | 89382735 |
| Sequence Number | 241637 | 85271 |
| Max. Length (bp) | 19992 | 19992 |
| Mean Length (bp) | 1306.46 | 1048.22 |
| N50 (bp) | 1880 | 1582 |
| N50 Sequence No. | 54262 | 16703 |
| N90 (bp) | 593 | 447 |
| N90 Sequence No. | 167717 | 60812 |
| GC% | 38.38 | 37.84 |


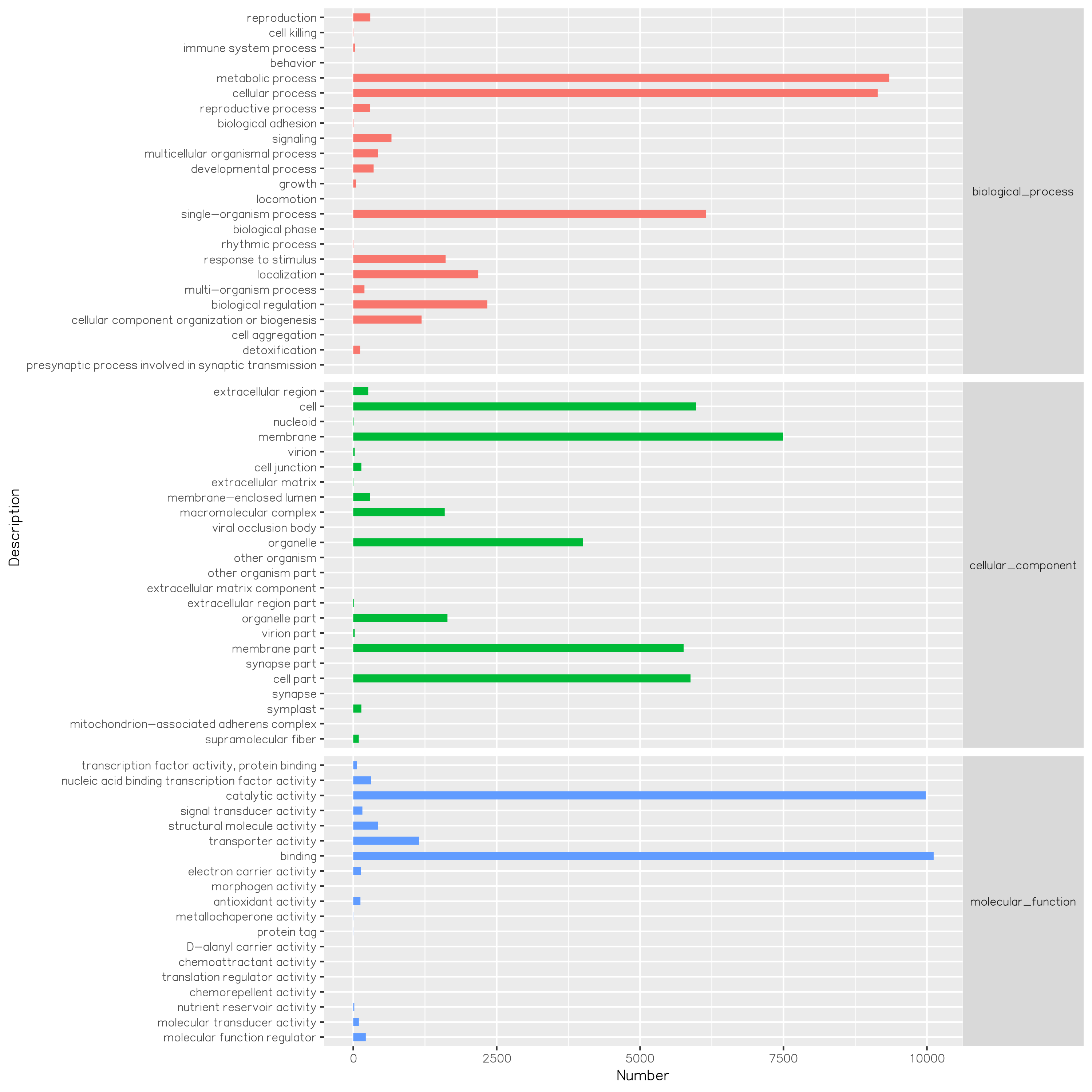


Supplementary Figure 5 The GO classification of the annotated genes.


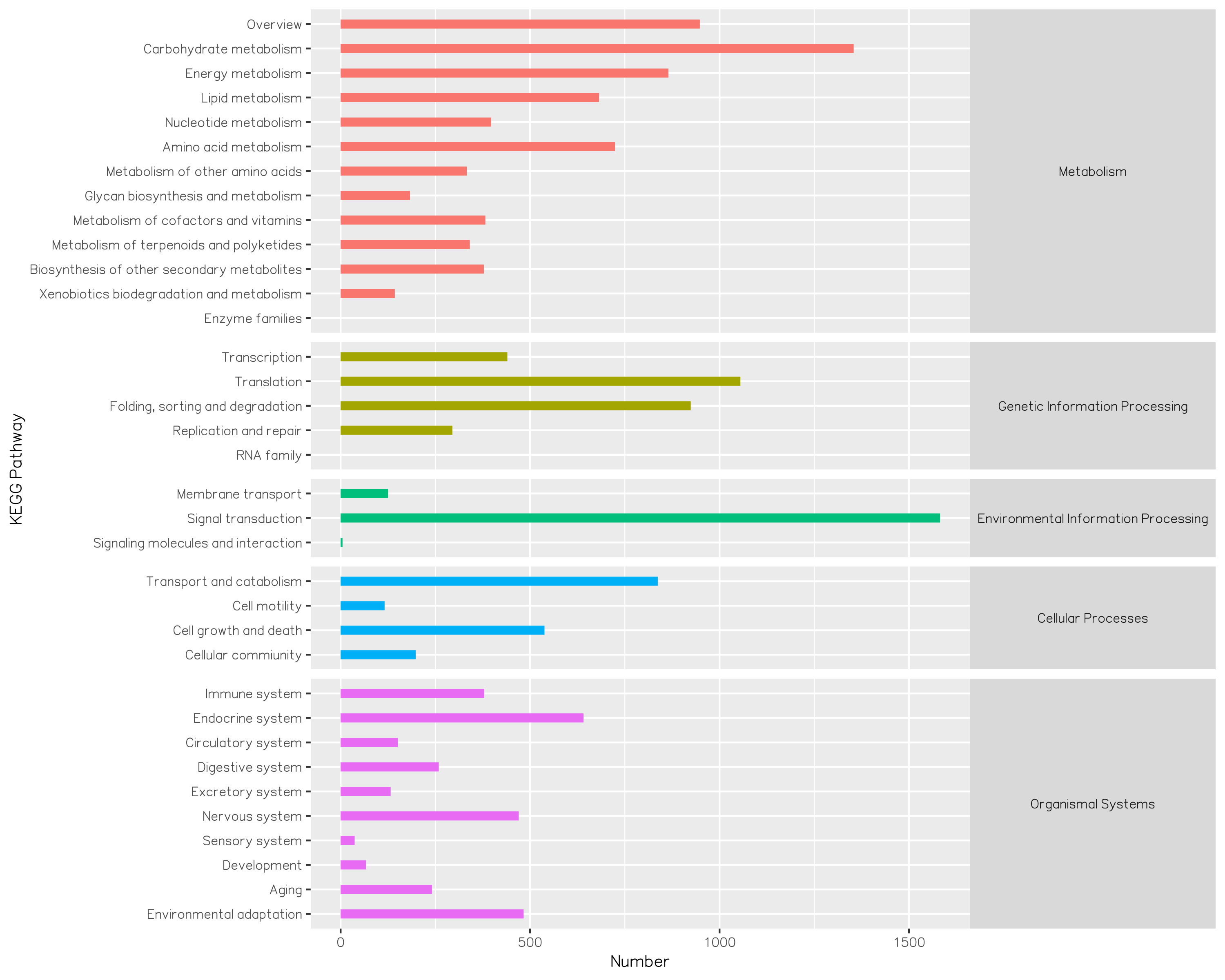


Supplementary Figure 6 The KEGG Pathway of the annotated genes.


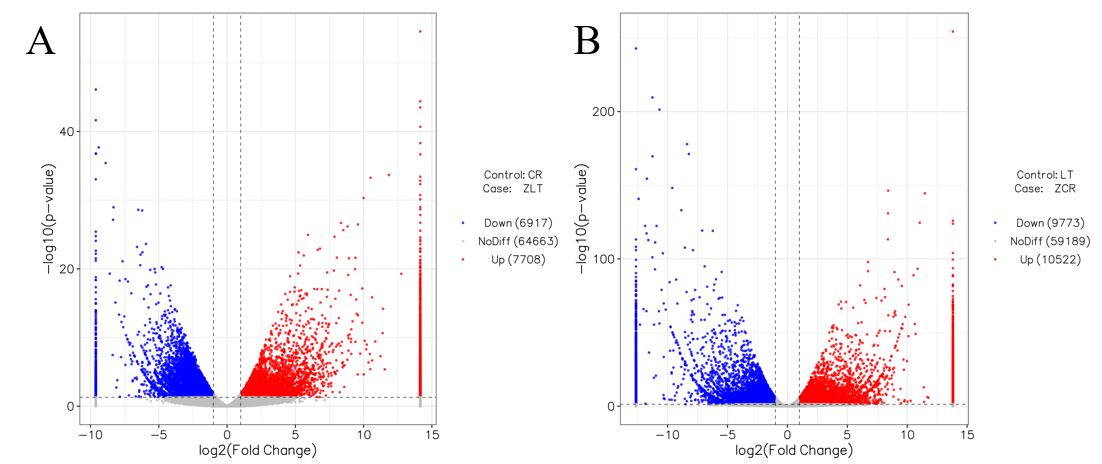


Supplementary Figure 7 Volcano map of DEGs. A: Volcano map of CR vs ZLT. B: Volcano map of LT vs ZCR.


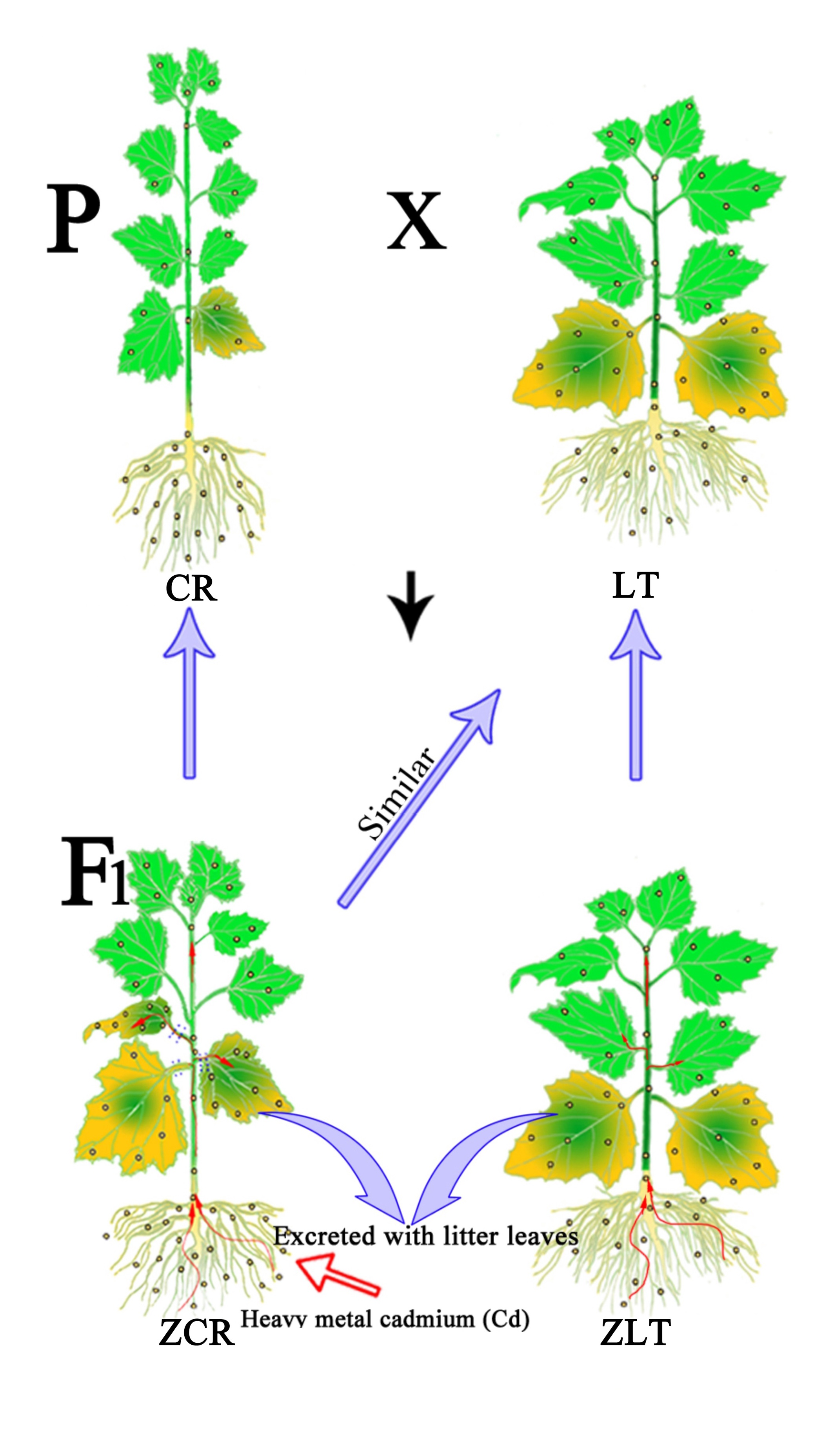


Supplementary Figure 8 Image Schema of PCA analysis of DEGs expression and Pot experiment data.


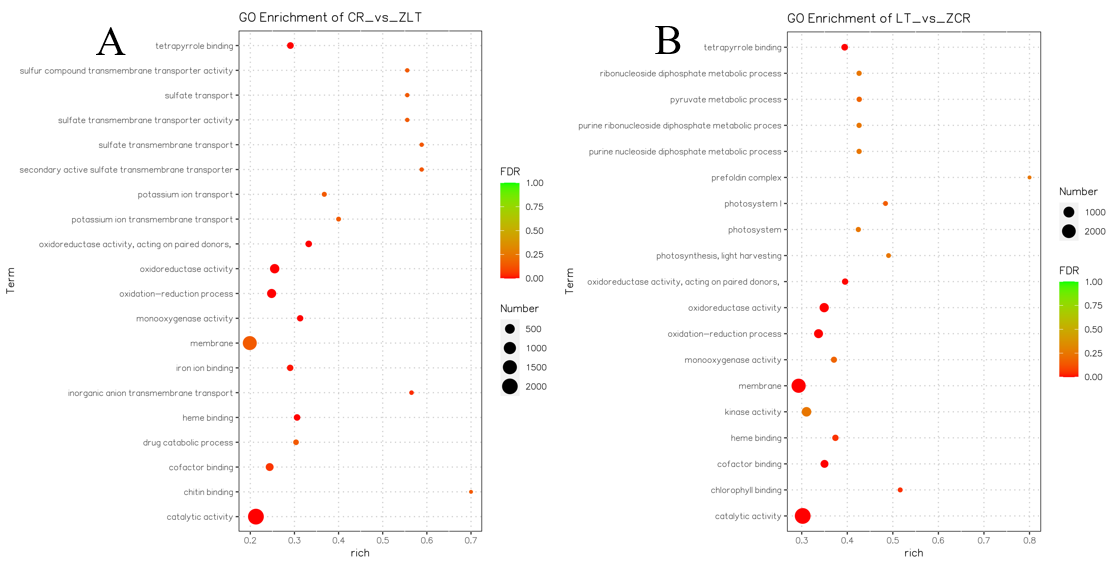


Supplementary Figure 9 GO enrichment of DEGs. A: GO enrichment of CR vs ZLT. B: GO enrichment of LT vs ZCR.


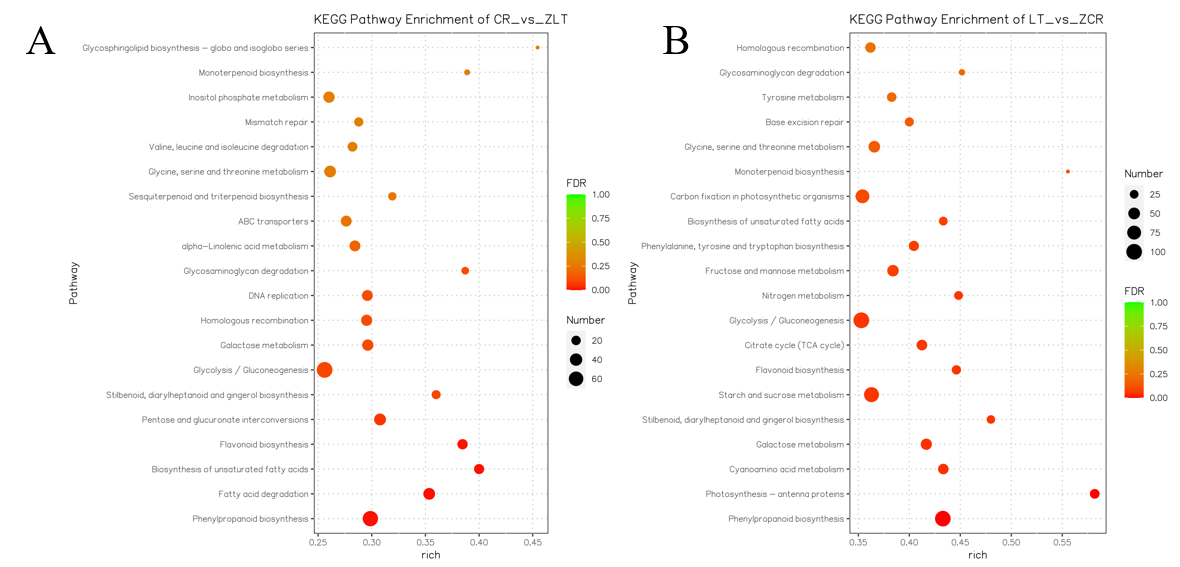


Supplementary Figure 10 KEGG enrichment of DEGs. A: KEGG enrichment of CR vs ZLT. B: KEGG enrichment of LT vs ZCR.


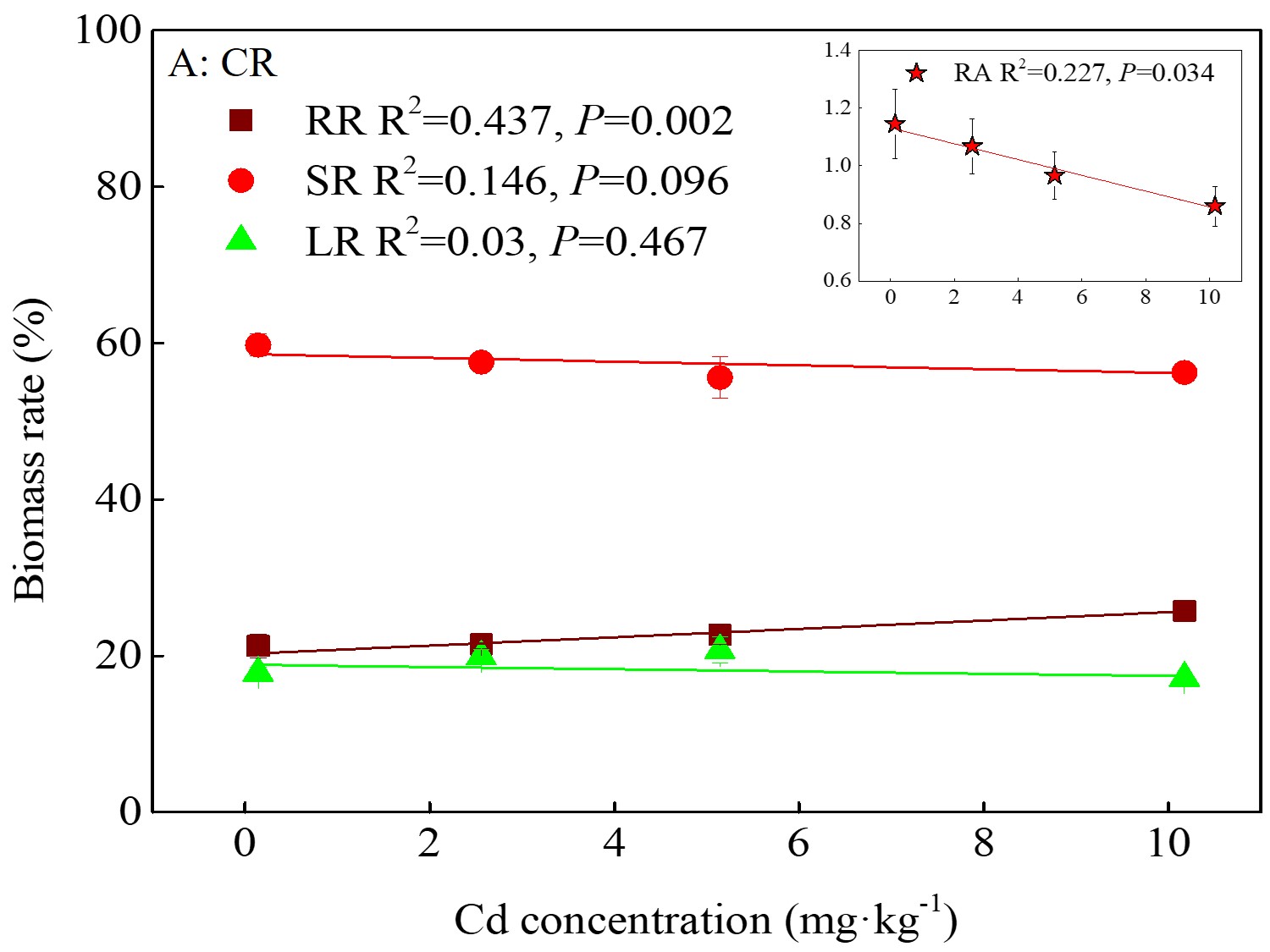

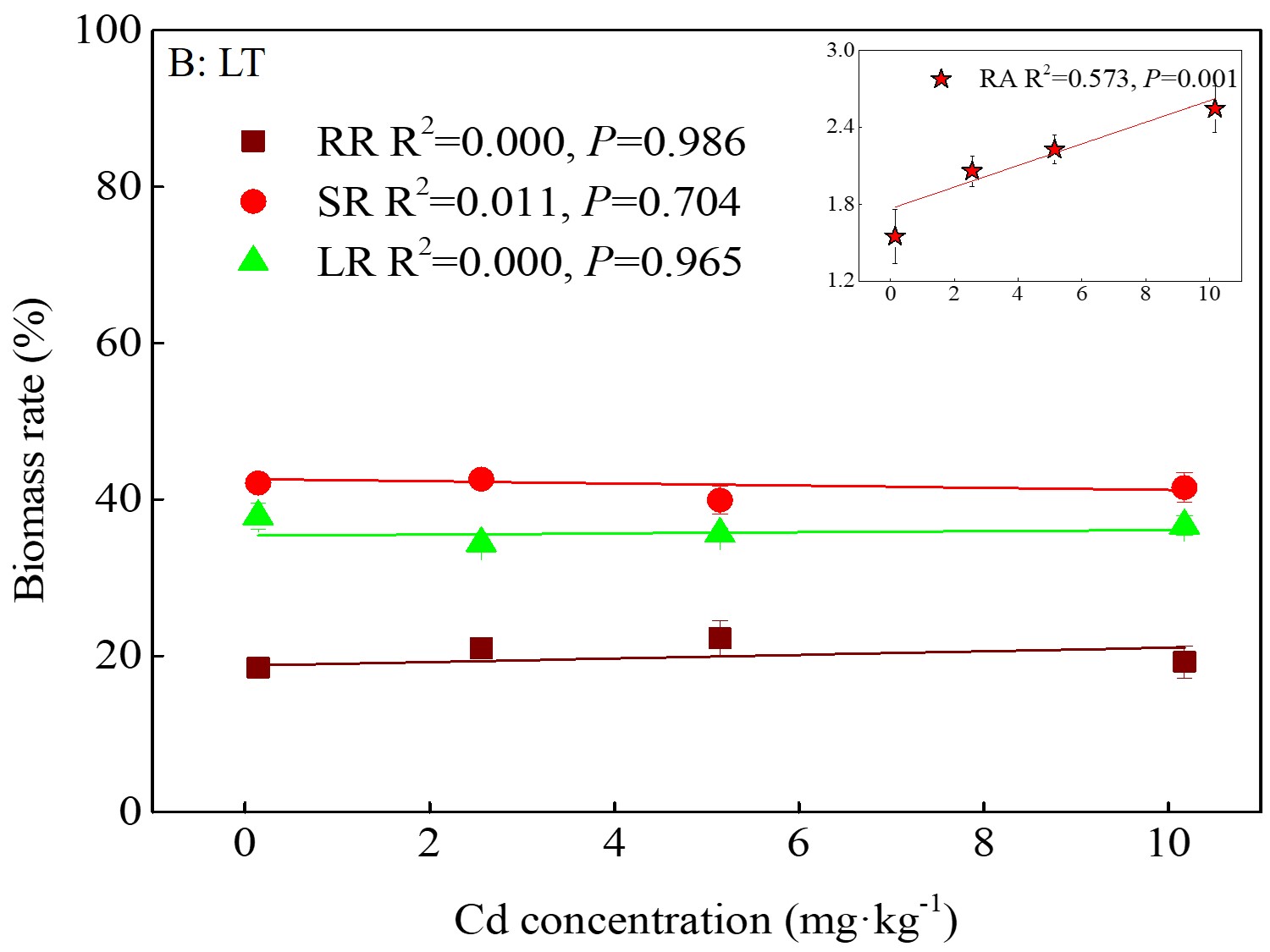


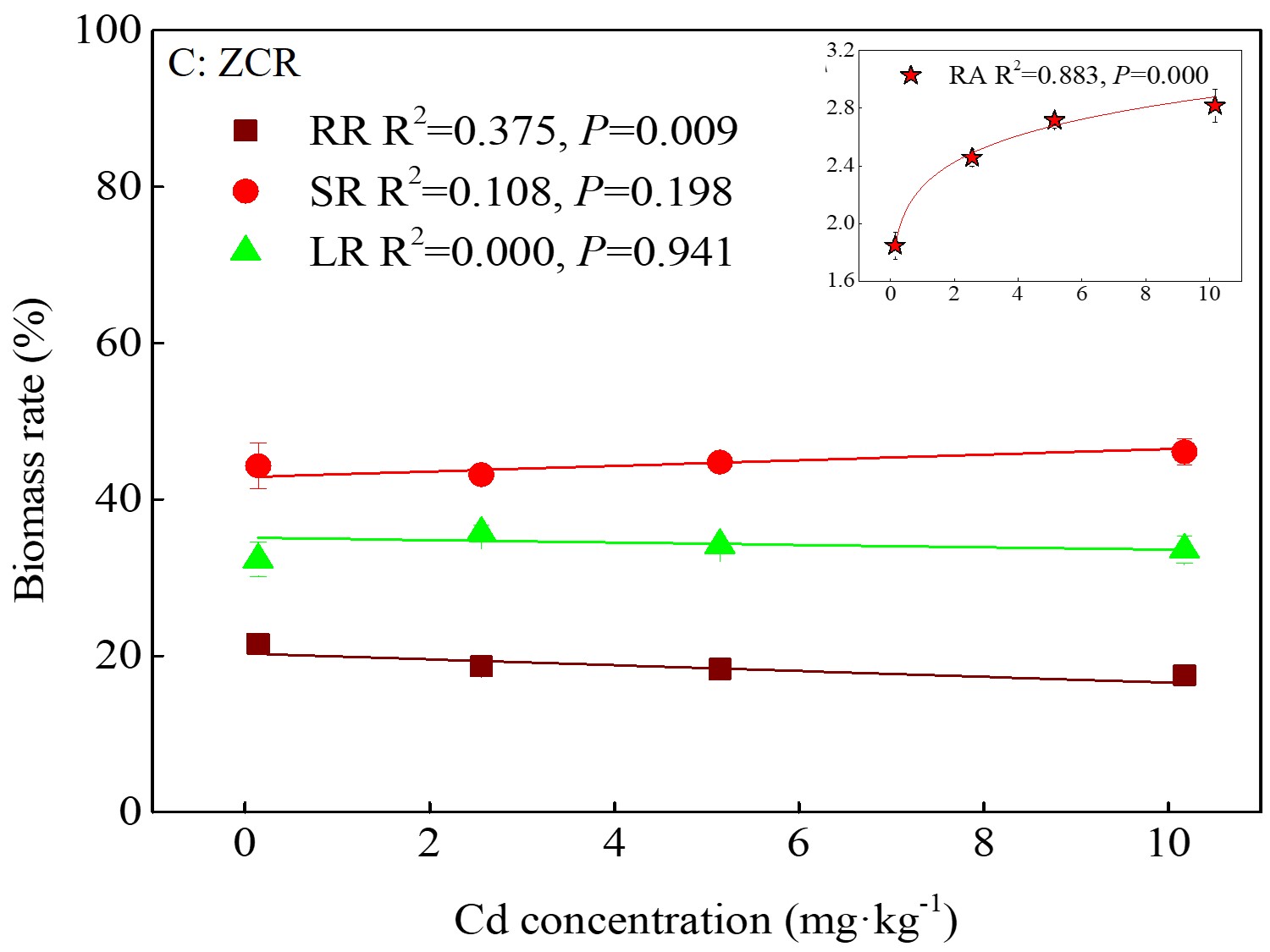

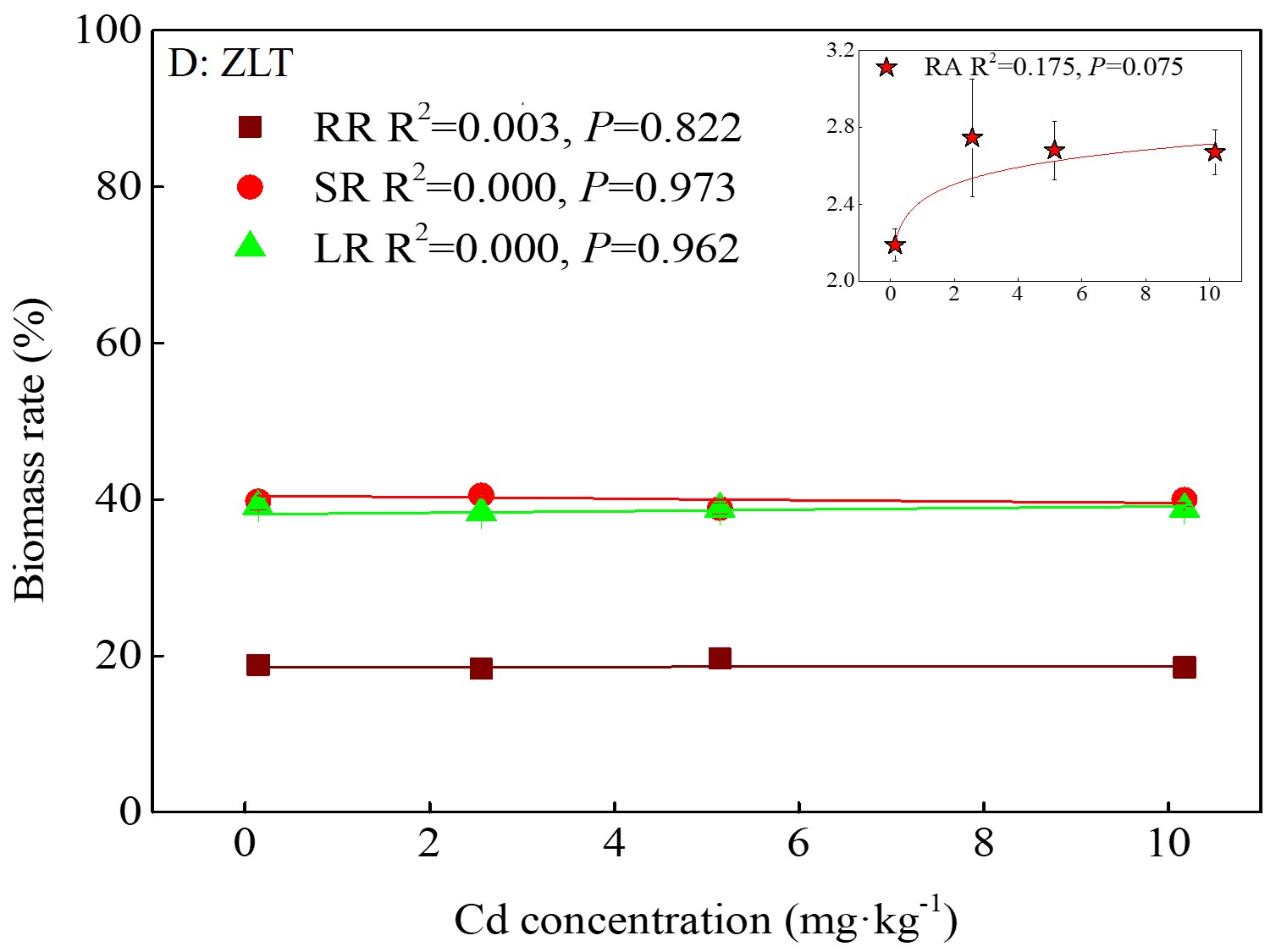


Supplementary Figure 11 Biomass ratio and Soil Cd treatment-response relationships of CR, ZCR, ZLT and LT. Root biomass ratio (RR). Stem biomass ratio (SR). Leave biomass ratio (LR). Reproductive allocation (RA).

Determination of photosynthetic index

The maximum net photosynthetic rate (*P*_max_) of plants were determined by using the Li-6800 portable photosynthesis device (Li-COR, Lincoln, NE, USA). The leaf chamber light intensity was set to 1500 µmol·m^-2^·s^-1^ (saturation light intensity is determined by measurement), and the reference chamber CO_2_ concentration was 385 µmol·mol^-1^, the leaf temperature was controlled at 28 °C, and the relative humidity was 59%.


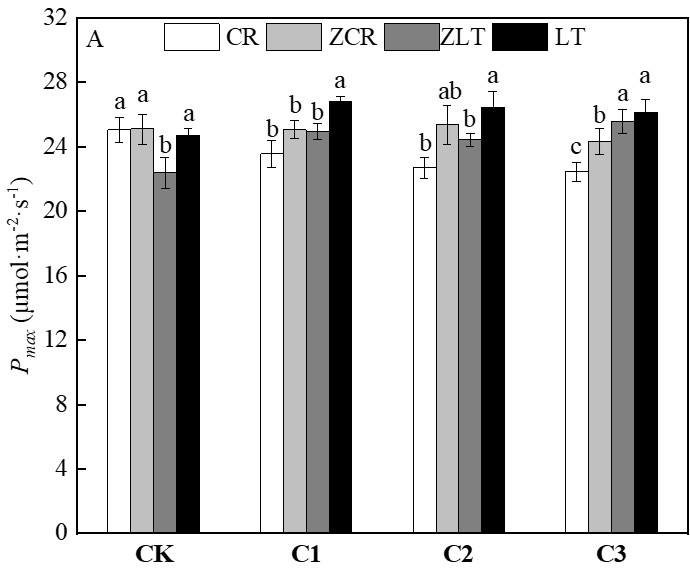

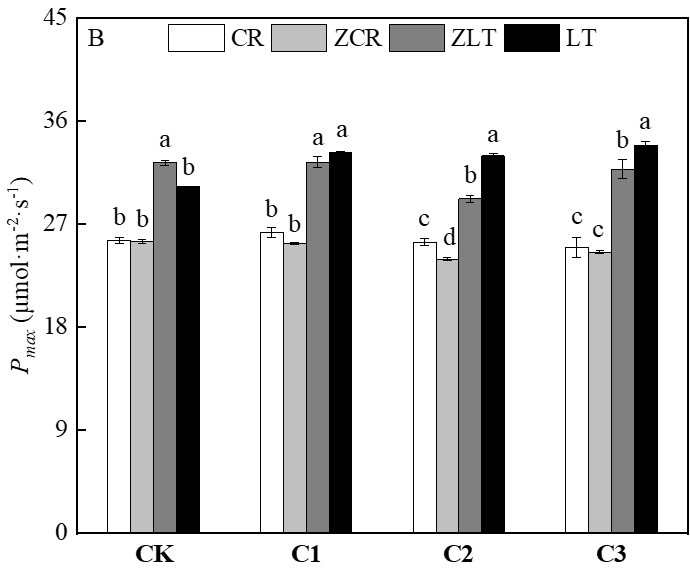


Supplementary Figure 12 *P*_max_ of CR, ZCR, ZLT and LT at different growth stages. A: *P*_max_ of CR, ZCR, ZLT and LT at vegetative growth stages. B: *P*_max_ of CR, ZCR, ZLT and LT at reproductive growth stages. Within the same Cd concentration, different small letters indicate significant differences in the same plants (*P*<0.05; *df*: between groups = 3, within groups = 20).

Determination of reproductive efficiency index

The reproductive efficiency index of the plant are calculated (Shen et al., 2016).

$$\text{Reproductive efficiency index }\left(\text{flower}\cdot\text{mg}^{\text{-1}}\right)=\frac{\text{flower number}}{\text{total biomass of each plant}}$$

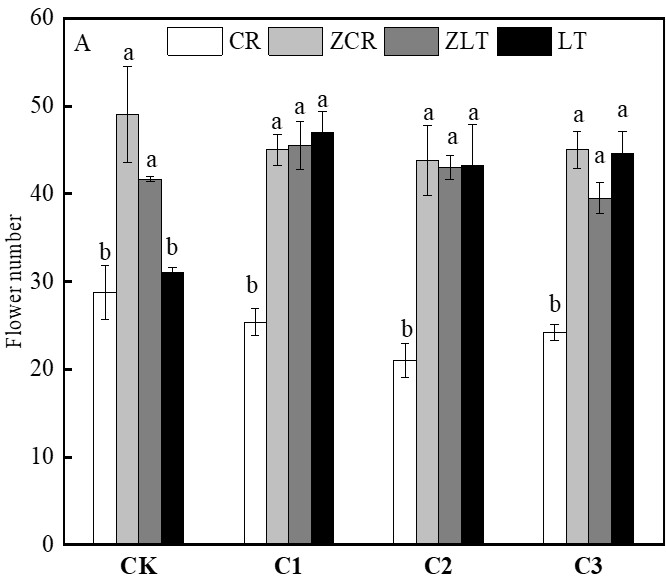

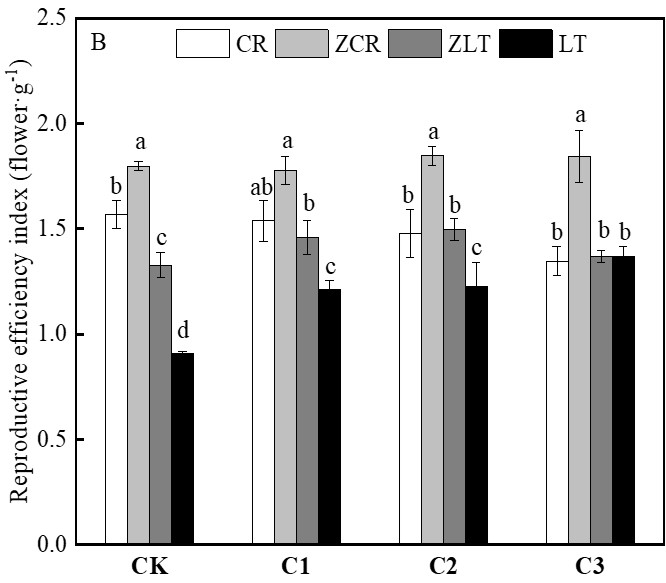


Supplementary Figure 13 The number of flower and reproductive efficiency index of CR, ZCR, ZLT and LT under different Cd treatment. A: The number of flower in CR, ZCR, ZLT and LT. B: The reproductive efficiency index of CR, ZCR, ZLT and LT. Different small letters indicate significant differences among CR, ZCR, ZLT and LT at the same treatment (*P*<0.05; *df*: between groups = 3, within groups = 20).
